# Transcriptional and Post-Transcriptional Regulation and Transcriptional Memory of Chromatin Regulators in Response to Low Temperature

**DOI:** 10.3389/fpls.2020.00039

**Published:** 2020-02-07

**Authors:** Kora Vyse, Léa Faivre, Melissa Romich, Majken Pagter, Daniel Schubert, Dirk K. Hincha, Ellen Zuther

**Affiliations:** ^1^Central Infrastructure Group Genomics and Transcript Profiling, Max-Planck-Institute of Molecular Plant Physiology, Potsdam, Germany; ^2^Institute of Biology, Freie Universität Berlin, Berlin, Germany; ^3^Department of Chemistry and Bioscience, Aalborg University, Aalborg East, Denmark

**Keywords:** chromatin regulators, quantitative reverse transcription polymerase chain reaction platform, histones, cold acclimation, deacclimation

## Abstract

Chromatin regulation ensures stable repression of stress-inducible genes under non-stress conditions and transcriptional activation and memory of stress-related genes after stress exposure. However, there is only limited knowledge on how chromatin genes are regulated at the transcriptional and post-transcriptional level upon stress exposure and relief from stress. We reveal that the repressive modification histone H3 lysine 27 trimethylation (H3K27me3) targets genes which are quickly activated upon cold exposure, however, H3K27me3 is not necessarily lost during a longer time in the cold. In addition, we have set-up a quantitative reverse transcription polymerase chain reaction-based platform for high-throughput transcriptional profiling of a large set of chromatin genes. We find that the expression of many of these genes is regulated by cold. In addition, we reveal an induction of several DNA and histone demethylase genes and certain histone variants after plants have been shifted back to ambient temperature (deacclimation), suggesting a role in the memory of cold acclimation. We also re-analyze large scale transcriptomic datasets for transcriptional regulation and alternative splicing (AS) of chromatin genes, uncovering an unexpected level of regulation of these genes, particularly at the splicing level. This includes several vernalization regulating genes whose AS may result in cold-regulated protein diversity. Overall, we provide a profiling platform for the analysis of chromatin regulatory genes and integrative analyses of their regulation, suggesting a dynamic regulation of key chromatin genes in response to low temperature stress.

## Introduction

Plants are exposed to a multitude of abiotic and biotic stresses during their lifetime and have evolved efficient mechanisms to cope with such events. Stress alleviation relies on numerous changes at the biochemical, physiological and molecular level, largely coordinated by a massive and fast reprogramming of the transcriptome. While it is known that the exposure to chilling temperatures results in the up- and downregulation of thousands of genes, as well as extensive post-transcriptional regulation of genes such as alternative splicing, all within minutes of cold exposure (Calixto et al., 2018), the precise mechanisms leading to this reprogramming have not been completely elucidated yet. Changes in the transcriptional activity of cold-stress-responsive genes upon exposure to low temperatures might be partly achieved through remodeling of the chromatin, rendering it more or less accessible for the transcriptional machinery, thereby affecting the expression levels of the genes. After cold stress ends and deacclimation begins, most stress-responsive genes return quickly back to their initial transcriptional levels (Byun et al., 2014; Pagter et al., 2017), suggesting that cold-induced changes to the chromatin might be reversed during deacclimation. Cold induced changes to the chromatin have in particular been studied in the context of vernalization. Vernalization is defined as the acquisition of the competence to flower after prolonged cold treatment, allowing vernalization-responsive plants to flower in spring, under favorable temperature conditions and the appropriate photoperiod. This process relies on epigenetic mechanisms, as cold induces a mitotically stable switch inhibiting the expression of the floral repressor *FLOWERING LOCUS C (FLC)*. The repression of *FLC* is achieved through the action of regulators of the Polycomb-group (Pc-G), which deposit and maintain the repressive tri-methylation of lysine 27 in histone H3 (H3K27me3) upon cold exposure (Song et al., 2012). Changes in the chromatin state were also previously described for cold-inducible genes not involved in vernalization, suggesting the involvement of dynamic chromatin regulation in the induction and repression of cold stress-responsive genes (Kwon et al., 2009; Park et al., 2018). Hyper-acetylation of histone H3K9 in promoter regions of *DREB1*, a main regulator of cold response, was observed in rice and was decreased again when plants were returned to control temperatures, suggesting that deacetylation keeps the gene in an off-state in the absence of cold (Roy et al., 2014). Recently, epigenetic changes involved in cold response were reviewed by different authors (Baulcombe and Dean, 2014; Kim et al., 2015; Asensi-Fabado et al., 2017; Banerjee et al., 2017; Luo et al., 2017; Friedrich et al., 2019). Furthermore, epigenetic changes that establish environmental memory in plants have been described (He and Li, 2018). The existence of memory of a cold priming event, including transcriptional memory, resulting in improved freezing tolerance after a subsequent triggering cold treatment was recently shown (Zuther et al., 2019).

In general, chromatin can be distinguished into euchromatin, consisting of mostly active genes and closed inactive heterochromatin, with a preference for repetitive elements (Allis and Jenuwein, 2016). Chromatin is composed of basic repeating units called nucleosomes, consisting of 145 to 147 bp of DNA wrapped around a histone octamer (Luger et al., 1997). The octamer is formed by core histones H2A, H2B, H3, and H4 and the repeating nucleosomal structure is further linked and stabilized by the linker histone H1 (Luger et al., 1997). This results in the arrangement of higher-order helical structures (Widom, 1989). The nucleosome not only helps in the packaging of the DNA, but is also the primary determinant of DNA accessibility (Luger et al., 1997). Histones, particularly their tails, are extensively post-translationally modified and can undergo a variety of covalent modifications such as acetylation, methylation, phosphorylation, and ubiquitination (Zhang and Reinberg, 2001).

Histone acetylation is set by histone acetyltransferases (HAC), which transfer the acetyl group of acetyl Coenzyme A to the ϵ-amino group of lysine side chains (Bannister and Kouzarides, 2011). Histone acetylation is associated with a function as a transcriptional coactivator by neutralizing the positive charge of the lysine. The modification is reversible and the acetyl group can be removed by histone deacetylases to restore the positive charge of the lysine, thus stabilizing the local chromatin architecture. Histone methylation predominantly occurs on the amino acids lysine and arginine. Unlike acetylation, the charge of the histone is not affected (Bannister and Kouzarides, 2011). Lysine residues can be mono-, di-, or trimethylated, while arginine can contain one or two methyl groups on its guanidinyl group (Ng et al., 2009). Histone methylation can lead to an active or repressive chromatin state, depending on the modified residue and the number of added methyl groups. Trithorax group (Trx-G) factors are responsible for the deposition of activating methylations on lysine 4 and 36 of histone 3 (H3K4me3 and H3K36me3, respectively), leading to the transcriptional activation of their target genes (Del Prete et al., 2015). Their action is antagonized by the proteins of the Polycomb Repressive Complex 2 (PRC2), which mediates H3K27me3. This highly conserved methyltransferase complex contains three orthologues of Enhancer of zeste [E(z); CURLY LEAF (CLF), SWINGER (SWN), and MEDEA (MEA)], three orthologues of Suppressor of zeste12 [Su(z)12; EMBRYONIC FLOWER2 (EMF2), VERNALISATION2 (VRN2), and FERTILISATION INDEPENDENT SEED2 (FIS2)], five orthologues of Multicopy Suppressor of Ira (MSI1-5), and a single copy of Extra Sex Combs (ESC) [FERTILIZATION INDEPENDENT ENDOSPERM (FIE) in *Arabidopsis thaliana* (Kleinmanns and Schubert, 2014). Methylation can be reversed by two different classes of histone demethylases: while LSD1-type demethylases can remove one of two methyl groups, JUMONJI-type histone demethylases can counteract mono-, di- or trimethylation (Shi et al., 2004). The second highly conserved Polycomb-Repressive Complex, PRC1, is a histone ubiquitination complex and monoubiquitylates histone H2A (Kleinmanns and Schubert, 2014).

In addition to histone modifications, the state of chromatin can also be affected by DNA methylation, which was found to be linked to gene repression (Hotchkiss, 1948; Razin and Riggs, 1980). DNA methylation (5-methyl cytosine) can be a heritable epigenetic mark (Jin et al., 2011) and is set by DNA methyltransferases. In plants, cytosine can be methylated symmetrically [CG and CHG methylation (where H is any base except G) as well as asymmetrically (CHH)] and these modifications are predominantly found on transposons and other repetitive DNA elements (Zhang et al., 2006). Small RNAs generated by RNA interference (RNAi) target genomic DNA sequences for cytosine methylation (RNA-directed DNA methylation) (Law and Jacobsen, 2010). DNA methylation is reversible and the methyl groups can be removed by demethylases (Penterman et al., 2007). In general, promoter DNA methylation is associated with the repressive state of chromatin, as it alters the accessibility for transcription factors (Carey et al., 2011).

Dynamic regulation of chromatin is not only achieved by enzymes setting and removing chemical modifications at DNA or histones, but also by replacement of the canonical histones by histone variants, resulting in an immediate loss of histone modifications and a resetting of epigenetic changes (Spiker, 1982). Although nucleosomes are energetically stable, histones can be turned over. Histones H2A and H2B can be exchanged much faster than histones H3 and H4 (Weber and Henikoff, 2014). In *A. thaliana*, 13 H2A variants (labeled HTA1-13) exist, and are clustered in four groups: H2A, H2A.X, H2A.W, and H2A.Z (Kawashima et al., 2015). Histone H3 exists in 15 variants (labeled HTR1-15) distributed in categories including H3.1, H3.3, and CenH3 (Stroud et al., 2012). Different histone variants carry different functions and are located at different parts of the gene to convey regulation. The H3.3 variants are predominantly located towards the 3′-ends of genes and are generally associated with elevation of gene expression, whereas H2A.W binds to heterochromatin with its C-terminal motif KSPKKA and promotes heterochromatin condensation (Yelagandula et al., 2014; Kawashima et al., 2015). Additionally studies have identified H4 variants, however, protein variants have not been described in *A. thaliana* (Kawashima et al., 2015). Lastly, three copies of linker histone H1 exist in *A. thaliana*, H1.1, H1.2, and H1.3 (Kotliński et al., 2016). H1.3 is a stress-inducible histone variant and might be responsible for regulating dynamic DNA methylation (Rutowicz et al., 2015).

While there is ample evidence for a role of chromatin remodeling in the regulation of gene expression in response to cold, relatively little is known about the involvement of specific chromatin regulators. The transcriptional and post-transcriptional regulation of the expression of most of these chromatin modifier genes both during and after cold exposure remains unexplored as well. In the case of vernalization, *VERNALIZATION INSENSITIVE 3* (*VIN3*) is the only VRN gene known to be induced by cold, however, protein level analyses of Pc-G proteins involved in vernalization suggest post-transcriptional regulation of several genes including *VRN2*, *CLF*, *FIE*, and *SWN* (Wood et al., 2006).

Here, we set out to analyze the transcriptional and post-transcriptional regulation of chromatin regulatory genes in response to cold stress and following deacclimation using both publically available datasets and generation of a quantitative reverse transcription polymerase chain reaction (RT-qPCR) platform. We identify a potential role for Pc-G proteins in repressing stress-inducible genes under non-stress conditions and substantial transcriptional and post-transcriptional regulation of chromatin regulatory genes.

Interestingly, genes involved in vernalization are largely not transcriptionally regulated under short-term (3 days) cold conditions. However, they may be alternatively spliced, resulting in potentially altered protein sequences. Based on data generated with the RT-qPCR platform, we have identified additional cold-inducible chromatin regulatory genes and genes specifically regulated during deacclimation, including DNA demethylases and histone variants.

## Methods

### Plant Material

*A. thaliana* accession Col-0 seeds were sown in a single pot and grown on soil in a climate chamber with 20°C day-time temperature and 6°C night-time temperature in a 14-h light cycle with a light intensity of 180 µE m^−2^ s^−1^ and a humidity of 60% at day and 70% at night. After 1 week the plants were moved to a short-day climate chamber with the following conditions: 20°C/16°C day/night, 8 h day length, 180 µE m^−2^ s^−1^, humidity of 60%/75% day/night. The plants were kept under these short-day conditions for a week before transferring them to new pots (10 plants per 10-cm diameter pot). Afterwards, the plants were kept for another 7 days under short-day conditions before transfer to long-day conditions for another week. The conditions for long-day were 20°C day and 16°C night temperature with a day length of 16 h at a light intensity of 200 µE m^−2^ s^−1^. These 4-week-old plants were used in cold acclimation and deacclimation experiments. For cold acclimation, plants were moved for 3 days to a growth chamber with a constant temperature of 4°C and a day length of 16 h with a light intensity of 90 µE m^−2^ s^−1^ and a humidity of 70% to 80% (Zuther et al., 2019). For deacclimation, plants were moved back to previous growth conditions for up to 24 h (Pagter et al., 2017). Plant material of 10 individual replicate plants was harvested from non-acclimated (NA) plants (at 8 am), after 3 days of cold acclimation (at 8 am) and after 2, 4, 6, 12, and 24 h of deacclimation (Deacc). The material was immediately frozen in liquid nitrogen and stored at −80°C before being ground into a fine powder in a ball mill (Retsch, Haan, Germany).

### Selection of Genes of Interest for the RT-qPCR Platform

We focused our selection on chromatin genes associated with epigenetic changes and selected 135 genes for analyses (see **Table 1** for abbreviations and **Supplemental Table 2** for primer sequences). These include Pc-G genes and Pc-G associated genes (and their paralogs), Trx-G genes, a selection of histone demethylase genes (putative H3K9 and H3K27 demethylases (JUMONJI-type) and LSD1-like histone demethylases), DNA methyltransferase and demethylase, and canonical histone and histone variant genes.

**Table 1:** Quantitative reverse transcription polymerase chain reaction (RT-qPCR) platform: Nomenclature of 135 genes encoding epigenetic regulators.

Locus ID	Abbreviation	Gene annotation
AT5G04240	JMJ11/ELF6	Probable lysine-specific demethylase ELF6, JMJ11
AT3G48430	JMJ12/REF6	Lysine-specific demethylase REF6, JMJ12
AT5G46910	JMJ13	Jumonji (jmj) family protein/zinc finger (C5HC2 type) family protein, JMJ13
AT1G08620	JMJ16	Transcription factor PKDM7D; JMJ16
AT2G38950	JMJ19	Jumonji and C5HC2 type zinc finger domain-containing protein, JMJ19
AT5G63080	JMJ20	HR demethylase JMJ20
AT1G78280	JMJ21	Transcription factor jumonji domain-containing protein, JMJ21
AT5G06550	JMJ22	HR demethylase-like protein, JMJ22
AT1G09060	JMJ24	JmjC domain protein JMJ24
AT3G07610	JMJ25/IBM1	IBM1, JMJ25
AT4G00990	JMJ27	Transcription factor jumonji (jmjC) domain-containing protein, JMJ27
AT4G21430	JMJ28	Transcription factor jumonji domain-containing protein, B160
AT1G62310	JMJ29	Transcription factor jumonji domain-containing protein, JMJ29
AT3G20810	JMJ30	Jumonji-C domain-containing protein 30 (JMJ30); JMJD5
AT3G45880	JMJ32	2-oxoglutarate (2OG) and Fe(II)-dependent oxygenase superfamily protein (JMJ32)
AT3G10390	FLD	Lysine-specific histone demethylase 1 homolog 3
AT1G62830	LDL1	Lysine-specific histone demethylase 1 homolog 3
AT3G13682	LDL2	Lysine-specific histone demethylase 1-like 2
AT4G16310	LDL3	LDL3 protein LSD1-like 3
AT5G51230	EMF2	EMBRYONIC FLOWER 2 (EMF2)
AT4G02020	SWN	SWINGER (SWN)
AT3G18990	VRN1	REDUCED VERNALIZATION RESPONSE 1 (VRN1)
AT4G16845	VRN2	REDUCED VERNALIZATION RESPONSE 2 (VRN2)
AT1G49480	RTV1	RELATED TO VERNALIZATION1 1 (RTV1)
AT2G30470	HSI2	HIGH-LEVEL EXPRESSION OF SUGAR-INDUCIBLE GENE 2 (HSI2)
AT4G32010	HSL1	HSI2-LIKE 1 (HSL1)
AT5G05610	AL1	ALFIN-LIKE 1 (AL1)
AT3G11200	AL2	ALFIN-LIKE 2 (AL2)
AT3G42790	AL3	ALFIN-LIKE 3 (AL3)
AT5G26210	AL4	ALFIN-LIKE 4 (AL4)
AT5G20510	AL5	ALFIN-LIKE 5 (AL5)
AT2G02470	AL6	ALFIN-LIKE 6 (AL6)
AT1G14510	AL7	ALFIN-LIKE 7 (AL7)
AT1G49950	TRB1	TELOMERE REPEAT BINDING FACTOR 1 (TRB1)
AT3G49850	TRB3	TELOMERE REPEAT BINDING FACTOR 3 (TRB3)
AT5G18620	CHR17	CHROMATIN REMODELING FACTOR17 (CHR17)
AT2G23380	CLF	Histone-lysine N-methyltransferase CLF
AT3G20740	FIE1	Polycomb group protein FERTILIZATION-INDEPENDENT ENDOSPERM
AT5G58230	MSI1	Histone-binding protein MSI1
AT2G16780	MSI2	WD-40 repeat-containing protein MSI2
AT4G35050	MSI3	WD-40 repeat-containing protein MSI3
AT2G19520	MSI4	WD-40 repeat-containing protein MSI4
AT4G29730	MSI5	WD-40 repeat-containing protein MSI5
AT3G23980	BLI	Protein BLISTER
AT3G03140	PWO1	PWO1
AT1G51745	PWO2	PWO2
AT3G21295	PWO3	PWO3
AT2G40930	UBP5	Ubiquitin-specific protease 5
AT5G22030	UBP8	Ubiquitin-specific protease 8
AT4G06634	YY1	AtYY1
AT5G44280	RING1A	Putative E3 ubiquitin-protein ligase RING1a
AT1G03770	RING1B	Putative E3 ubiquitin-protein ligase RING1b
AT1G06770	DRIP1/BMI1B	E3 ubiquitin protein ligase DRIP1
AT2G30580	DRIP2/BMI1A	E3 ubiquitin protein ligase DRIP2
AT5G57380	VIN3	Protein VERNALIZATION INSENSITIVE 3
AT4G30200	VEL1	Vernalization5/VIN3-like protein
AT2G18880	VEL2	Vernalization5/VIN3-like protein
AT2G18870	VEL3	Vernalization5/VIN3-like protein
AT3G24440	VRN5	Protein VERNALIZATION 5
AT2G31650	ATX1	HOMOLOGUE OF TRITHORAX (ATX1)
AT1G05830	ATX2	TRITHORAX-LIKE PROTEIN 2 (ATX2)
AT3G61740	ATX3	ATX3
AT4G27910	ATX4	ATX4
AT5G53430	ATX5	ATX5
AT4G28190	ULT1	ULTRAPETALA1 (ULT1)
AT2G20825	ULT2	ULTRAPETALA 2 (ULT2)
AT3G06400	CHR11	Chromatin-remodeling protein 11
AT3G06010	CHR12	SNF2/Brahma-type chromatin-remodeling protein CHR12
AT5G14170	CHC1	Chromodomain remodeling complex protein CHC1
AT1G65470	FAS1	Chromatin assembly factor 1 subunit FAS1
AT5G64630	FAS2	Chromatin assembly factor 1 subunit FAS2
AT1G79350	FGT1	FORGETTER1
AT5G47690	PDS5a	PDS5a
AT4G31880	PDS5c	PDS5c
AT1G80810	PDS5d	PDS5d
AT1G15940	PDS5e	PDS5e
AT2G47620	SWI3A	SWITCH/SUCROSE NONFERMENTING 3A (SWI3A)
AT2G33610	SWI3B	SWITCH SUBUNIT 3 (SWI3B)
AT1G21700	SWI3C	SWITCH/SUCROSE NONFERMENTING 3C (SWI3C)
AT2G28290	SYD	Chromatin structure-remodeling complex protein SYD
AT2G46020	BRM	ATP-dependent helicase BRAHMA
AT5G04990	SUN1	SAD1/UNC-84 domain protein 1
AT3G10730	SUN2	SAD1/UNC-84 domain-containing protein 2
AT1G67230	CRWN1	LITTLE NUCLEI1
AT1G13220	CRWN2	LITTLE NUCLEI2
AT1G68790	CRWN3	LITTLE NUCLEI3
AT5G65770	CRWN4	LITTLE NUCLEI4
AT2G27100	SE	SERRATE (SE)
AT1G48410	AGO1	Protein argonaute 1
AT2G27040	AGO4	Argonaute 4
AT1G01040	DCL1	Endoribonuclease Dicer-like 1
AT3G03300	DCL2	Endoribonuclease Dicer-like 2
AT3G43920	DCL3	Endoribonuclease Dicer-like 3
AT5G20320	DCL4	Dicer-like protein 4
AT5G39550	ORTH1/VIM3	VARIANT IN METHYLATION 3 (VIM3)
AT1G57820	ORTH2/VIM1	E3 ubiquitin-protein ligase ORTHRUS 2
AT4G19020	CMT2	Chromomethylase 2
AT1G69770	CMT3	DNA (cytosine-5)-methyltransferase CMT3
AT5G15380	DRM1	DNA (cytosine-5)-methyltransferase DRM1
AT5G14620	DRM2	DNA (cytosine-5)-methyltransferase DRM2
AT3G17310	DRM3	Domains Rearranged Methyltransferase3
AT5G49160	MET1	DNA (cytosine-5)-methyltransferase 1
AT5G04560	DME	Transcriptional activator DEMETER
AT2G36490	DML1/ROS1	Protein ROS1
AT3G10010	DML2	Putative DNA glycosylase
AT4G34060	DML3	DEMETER-like protein 3
AT5G64610	HAM1	MYST family histone acetyltransferase 1
AT5G09740	HAM2	MYST family histone acetyltransferase 2
AT5G03740	HD2C	Histone deacetylase 2C
AT3G44750	HDA3	Histone deacetylase 3
AT4G16420	ADA2B	Transcriptional adapter ADA2b
AT1G06760	H1.1	Histone H1.1
AT2G30620	H1.2	Histone H1.2
AT2G18050	H1.3	Histone H1.3
AT5G54640	HTA1	H2A.1; Histone superfamily protein
AT4G27230	HTA2	Histone H2A 2
AT5G59870	HTA6	Histone H2A 6
AT5G27670	HTA7	Histone H2A protein 7
AT2G38810	HTA8	Histone H2A 8
AT1G52740	HTA9	Histone H2A protein 9
AT1G51060	HTA10	Histone H2A 10
AT3G54560	HTA11	Histone H2A protein 11
AT5G02560	HTA12	Histone H2A protein 12
AT3G20670	HTA13	Histone H2A 13
AT5G65360	HTR1	H3.1/HTR1; Histone superfamily protein
AT1G09200	HTR2	Histone H3
AT4G40030	HTR4	Histone H3.3
AT4G40040	HTR5	Histone H3
AT1G13370	HTR6	HTR6
AT5G10980	HTR8	Histone H3.3
AT5G65350	HTR11	Histone 3 11
AT1G01370	HTR12	Histone H3-like centromeric protein HTR12
AT5G10390	HTR13	Histone H3
AT1G75600	HTR14	Histone H3-like 3
AT5G12910	HTR15	Histone H3-like 4, HTR15

### Chromatin Immunoprecipitation and (RT-)qPCR Analysis

Plants were grown for 21 days on ½ MS medium under SD conditions (8 h light, 16 h darkness) and transferred to 4°C for 3 h or 3 days. Seedlings (0.5–1 g) were crosslinked in phosphate-buffered saline (PBS) containing 1% paraformaldehyde for 10 min under vacuum. The crosslink reaction was stopped by the addition of 0.125 M glycine. Samples were rinsed with ice-cold PBS solution and frozen in liquid nitrogen. Chromatin extraction was performed as previously described (Kleinmanns et al., 2017). The extracted chromatin was incubated rotating at 4°C for 16 h with 1 µg of antibodies (anti-H3K27me3, C15410195 Diagenode; anti-H3pan, C15200011 Diagenode; anti-IgG, C15410206 Diagenode). Twenty microliters of Protein A couple beads (Thermo Fisher Scientific) were added and the samples were incubated rotating at 4°C for 4 h. Beads were washed and DNA purified as previously described (Kleinmanns et al., 2017). The qPCR analysis was performed using the Takyon ROX SYBR MasterMix blue dTTP kit and the QuantStudio5 (Applied Biosystems). Primers were designed to amplify loci carrying H3K27me3 according to the Jacobsen Epigenome Browser (Zhang et al., 2007) and their sequences can be found in **Supplemental Table 1**. Amplification values were normalized to input and to the *FUSCA3* (AT3G26790) locus or the *ACTIN7* (AT5G09810) locus, depending on the analyzed mark. Statistical significance was tested using one-way ANOVA followed by a Dunnett's multiple comparison test using GraphPad Prism7.

For gene expression analysis, RNA was isolated from 100 mg of seedlings grown as described above, using the innuPREP Plant RNA Kit (Analytik Jena). RNA was quantified using a Nanodrop-1000 spectrophotometer (Thermo Scientific). Genomic DNA was removed using the DNase I kit (Thermo Scientific) and cDNA was synthetized using the RevertAid Reverse Transcriptase (Thermo Scientific). The quantitative PCR was performed as described above. The Ct values were normalized by subtracting the mean of the three housekeeping genes *ACTIN2* (AT3G18780), *PDF* (AT1G13320), and *TIP41* (AT4G34270) AT4G34270 and taken as the negative exponent to the base 2. Primer sequences can be found in **Supplemental Table 1**. Statistical significance was tested using one-way ANOVA followed by a Dunnett's multiple comparison test using GraphPad Prism7.

### RT-qPCR Analysis for the Epigenetic Primer Platform

RNA was isolated from a pool of five samples consisting of 10 different plants using Trizol reagent (BioSolve BV). RNA was quantified using a NanoDrop-1000 spectrophotometer (Thermo Scientific) and DNA was removed from the samples using a RapidOut DNA Removal Kit (Thermo Scientific). The absence of genomic DNA was tested by qPCR with intron-specific primers (Intron MAF5 AT5G65080) (Zuther et al., 2012). cDNA was synthesised by SuperScript IV Reverse Transcriptase (Invitrogen) and oligo dT18 primers. The quality of the cDNA was tested using primers amplifying the 3′ and 5′ region of *GAPDH* (AT1G13440) (Zuther et al., 2012). cDNA of three independent biological replicates for each time point was used for expression analysis (Pagter et al., 2017).

RT-qPCR was performed for 135 genes of interest (**Supplemental Table 2**) as previously described (Le et al., 2015). Expression of four housekeeping genes, *Actin2* (AT3G18780), *EXPRS* (AT2g32170), *GAPDH* (AT1G13440), and *PDF* (AT1G13320) was measured for each sample on each plate (**Supplemental Table 3**; compare Zuther et al., 2012). The Ct values were normalized by subtracting the mean of the four housekeeping genes from the Ct value of each gene of interest (ΔCt). Transcript abundance was expressed as 2^−ΔCt^. The log2 fold change of the normalized Ct values was calculated either relative to the values obtained from NA or cold-acclimated (ACC) plants.

Heat maps were constructed in RStudio (R Core Team, 2013; RStudio Team, 2016) using the pheatmap package version 1.0.12.

### Primer Design

Primers were either designed in Primer3 or taken from the literature as indicated in **Supplemental Table 2**. The specifications of the primers were: primer length 20–24 bases, amplicon size 60–150 bp, primer melting temperature 64°C ± 3°C, amplicon melting temperature 75°C–95°C, G/C content 45%–55%, maximum repetition of a nucleotide of 3, and a G/C clamp of 1. Primers with a binding site near the 3′ end of the respective gene were preferred. For highly homologous genes maximum primer length was increased to 30 bp.

### Bioinformatic Analyses of the Overlap of Cold-Regulated Genes and H3K27me3 Targets Using Public Data

The dataset of differentially expressed cold-regulated genes was extracted from previously published data (Calixto et al., 2018). Experimental conditions used in this publication were as follows. *A. thaliana* Col-0 plants were stratified for 4 days at 4°C in the dark and then grown in hydroponic culture for 5 weeks. Growth conditions were 12 h light (150 μE m^−2^ s^−1^)/12 h dark with a constant temperature of 20°C. The 4°C cold treatment was started at dusk. Rosette material was harvested and pooled per sampling point.

H3K27me3 targets were extracted from previously published data (Lafos et al., 2011). In this study, H3K27me3 targets genes were identified with whole genome tiling arrays using undifferentiated meristematic cells from the shoot and differentiated leaf tissue from *clavata3* mutant plants (Col-0 background). For this analysis the leaf samples were taken from plants grown for 9 weeks under short day conditions (8 h light/16 h dark).

The datasets were compared using the conditional formatting and filter function from Excel.

### Bioinformatic Analyses of the Cold Regulation of Chromatin Modifier Genes Using Public Data

For the investigation of the regulation of chromatin genes during cold exposure, a list of chromatin regulator genes was extracted from the Chromatin Database (ChromDB) (version of the 30^th^ of July, 2011) (Gendler et al., 2008). This list was overlapped with the lists of genes either differentially expressed or differentially spliced at any time point during cold exposure (Calixto et al., 2018) using the VennDiagram R package.

The analysis of the cold regulation of vernalization actors was performed by overlapping a list of genes involved in vernalization regulation with the lists of cold-regulated and cold-alternatively spliced genes previously described. The Venn diagram was plotted using the VennDiagram R package. The expression profiles of genes showing differential splicing upon cold exposure were generated and downloaded using the webservice at https://wyguo.shinyapps.io/atrtd2_profile_app/ (Zhang et al., 2017; Calixto et al., 2018). The translations of the differential usage transcripts were extracted from AtRTD2 (Zhang et al., 2017) and aligned to one another using the Needle algorithm (Madeira et al., 2019) to identify potential differences in amino acid sequences caused by cold exposure. The alignments were visualized using Multiple Align Show from the Sequence Manipulation Suite (Stothard, 2000).

### Statistics

The statistical significance of overlaps between different groups of genes was calculated using http://nemates.org/MA/progs/overlap_stats.html. The significance of gene expression changes was analysed using an unpaired two-sided t-test, performed in RStudio (R Core Team, 2013; RStudio Team, 2016). The significance levels are presented as followed: *, 0.05 > p > 0.01; **, 0.01 > p > 0.001; and ***, p < 0.001.

## Results

### Enrichment of H3K27me3 Target Genes in Early, but not Late Cold-Inducible Genes

**Figure 1:**
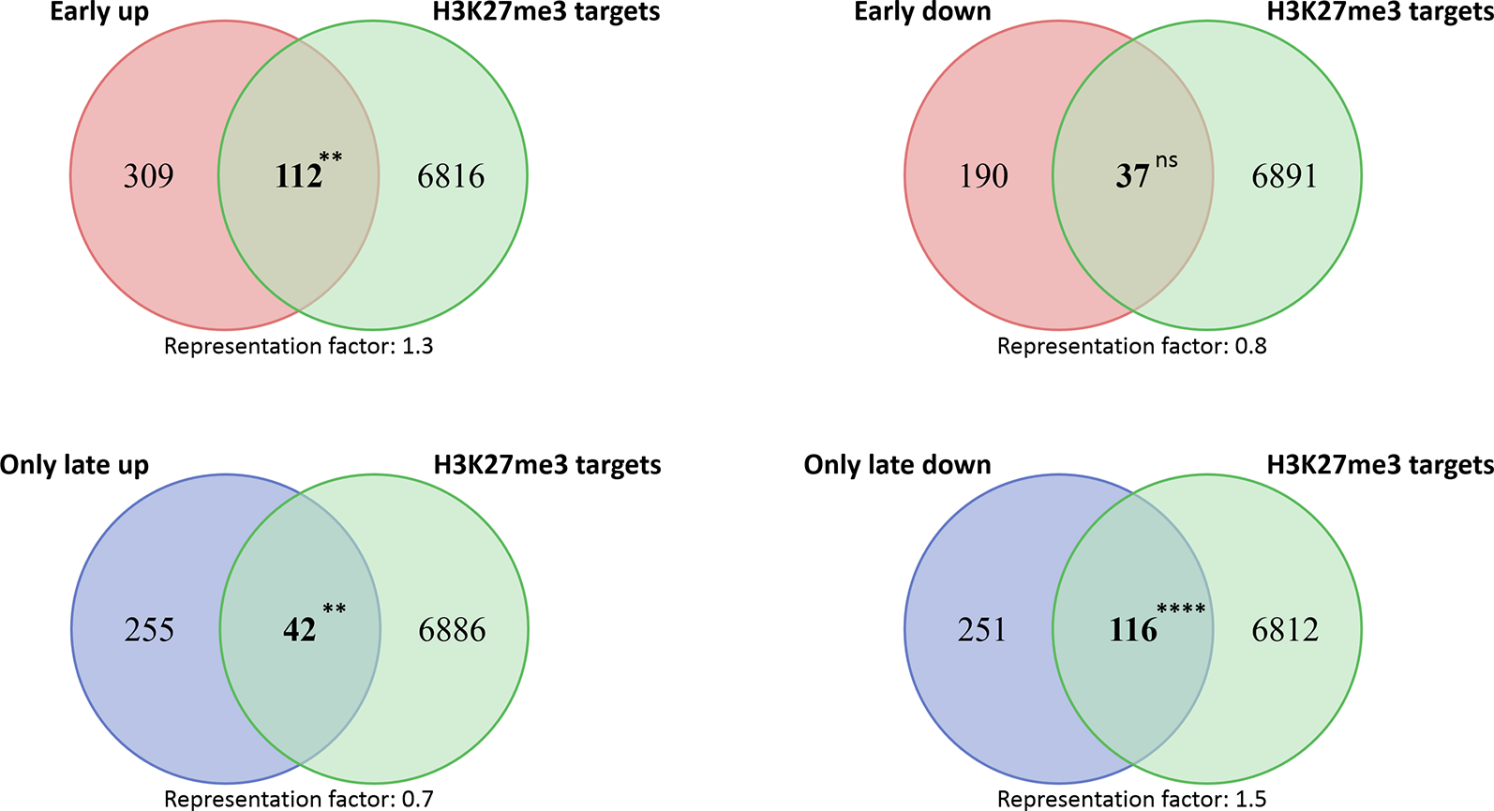
Venn diagrams showing the overlap of cold-regulated genes with H3K27me3 target genes. Four different cold-regulated gene groups, differing in their expression profile were extracted from Calixto et al. (2018). Genes already induced or repressed after 3 h in the cold are found in the group “Early up” or “Early down”, respectively. The “Only late” genes reflect genes which are not differentially expressed at 3 and 6 h and are induced or repressed at least six out of nine time points on day 3 in the cold (72 to 96 h). H3K27me3 targets were extracted from Lafos et al., 2011. Asterisks indicate ****P < 0.0001; **P < 0.01; ns stands for “not significant” and representation factor is given under each Venn diagram. For details see **Supplemental Table 4**.

Expression of stress-inducible genes needs to be tightly controlled to prevent costly induction of plant defense responses in the absence of abiotic and biotic stresses. We hypothesized that stress-inducible genes are epigenetically silenced under non-stress conditions and therefore analyzed the prevalence of the main epigenetic silencing mark H3K27me3 in cold-regulated genes. We bioinformatically compared the H3K27me3 target genes identified in mature leaves (profiled under ambient temperature conditions) with genes regulated quickly after cold exposure (3 h) or after long-term (3 d) cold (Lafos et al., 2011; Calixto et al., 2018) (**Figure 1**, **Supplemental Table 4**). H3K27me3 target genes were significantly enriched among the early (3 h) cold-inducible genes, but not in later (3 d) inducible genes. The opposite pattern was revealed for genes downregulated in the cold: H3K27me3 target genes are underrepresented in the early repressed genes, but enriched in the late repressed genes (3 d).

**Figure 2:**
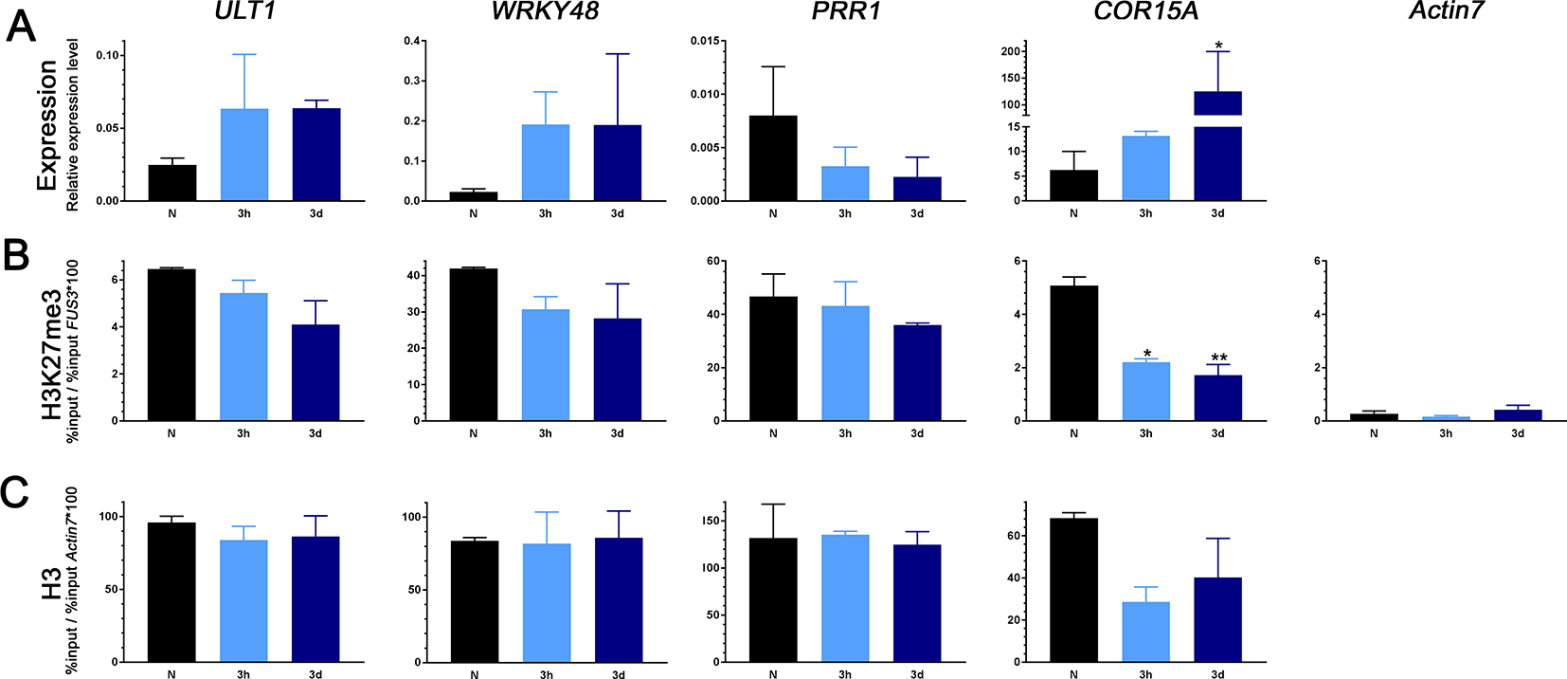
Gene expression changes, H3K27me3, and H3 levels of early upregulated (*ULT1*, *WRKY48*) and late downregulated (*PRR1*) genes as well as a well-characterized cold marker gene (*COR15A*) in plants exposed to cold. **(A)** RNA was isolated from 3-week-old seedlings grown at ambient temperature (N) and exposed to 4°C for 3 h or 3 days. Transcript levels for *ULT1*, *WRKY48*, *PRR1*, and *COR15A* were measured by reverse transcription and quantitative polymerase chain reaction (PCR). *ACTIN2*, *PDF*, and AT4G34270 were used as internal control. Error bars indicate ± s.e.m., n = 3 biological replicates. Test for significance by one-way ANOVA followed by a Dunnett's multiple comparison test. Significance levels are indicated relative to N: *P < 0.05. ChIP-PCR analysis of H3K27me3 **(B)** and H3 **(C)** occupancy. Chromatin was extracted from the same batch of seedlings used for RT-PCR after cross-linking and precipitated using H3K27me3 and H3 antibodies, respectively. The purified DNA was amplified by quantitative PCR. Results are presented as %input * 100/%input at the *FUS3* locus for H3K27me3 and at the *ACTIN7* locus for H3. For H3K27me3, *ACTIN7* was used as a negative control. Error bars indicate ± s.e.m, n = 2 biological replicates. Test for significance by one-way ANOVA followed by a Dunnett's multiple comparison test. Significance levels are indicated relative to N: *P < 0.05; **P < 0.01. All primer sequences used for this experiment can be found in **Supplemental Table 1**. Additional analyzed genes and %input values are shown in **Supplemental Figure 1**.

To reveal whether early activation or late repression of genes is associated with changes in H3K27me3 or H3 occupancy we analyzed several cold-inducible H3K27me3 target genes for their occupancy in ambient temperature and during cold exposure (3 h and 3 d) using chromatin immunoprecipitation (**Figure 2**, **Supplemental Figure 1**). While we confirmed a previous study showing that *COR15a* displayed a reduction in H3K27me3, possibly due to reduction in general occupancy of H3, after cold exposure (**Figure 2**; Kwon et al., 2009), the level of H3K27me3 (or H3) was not significantly altered at the cold inducible genes *ULTRAPETALA1* (*ULT1*), *WRKY48* (**Figure 2**), *TOLERANT TO CHILLING AND FREEZING1* (*TCF1*), and the glutaredoxin *GRXS4* (**Supplemental Figure 1**). Similarly, the late repressed gene *PINORESINOL REDUCTASE1* (*PRR1*) showed similar H3K27me3 (and H3) in ambient and cold temperatures (**Figure 2**). Thus, a change in cold-regulated gene expression is not necessarily associated with changes in H3K27me3 occupancy, at least at the analyzed genic regions.

### Transcriptional Regulation and Alternative Splicing of Chromatin Genes During Cold Exposure

**Figure 3:**
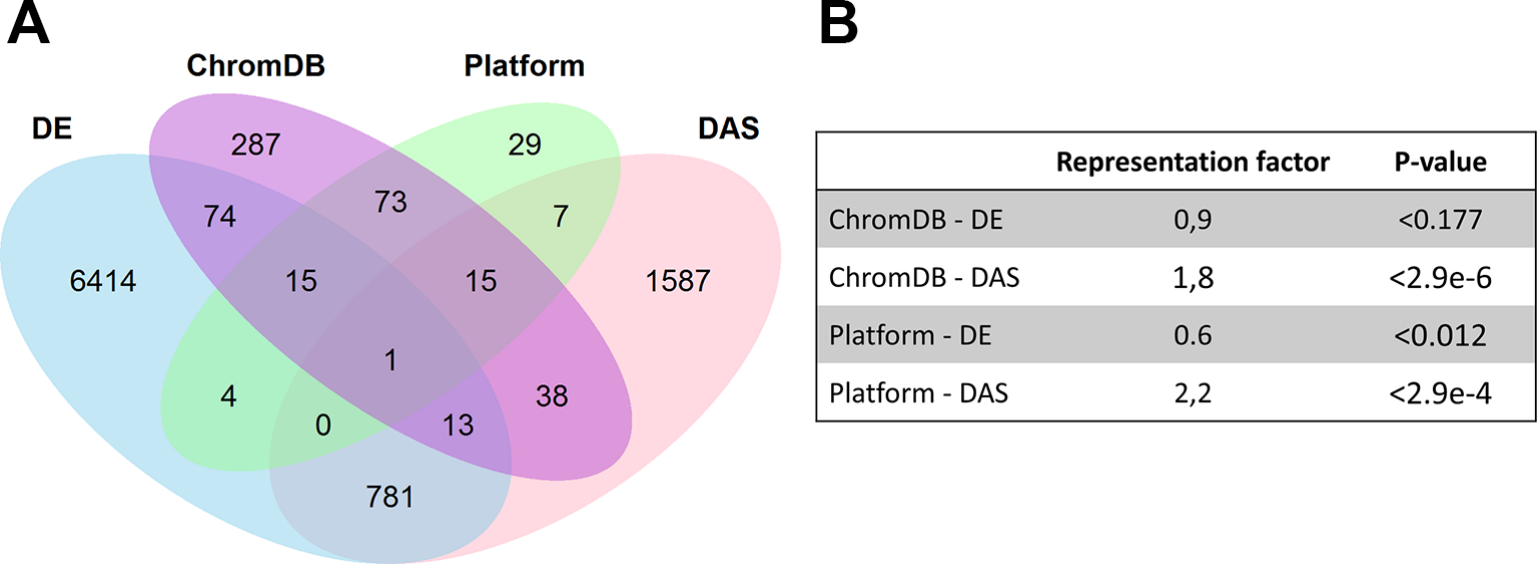
**(A)** Venn diagrams showing the overlaps of cold-regulated genes with chromatin regulatory genes. The overlaps were generated for genes extracted from the Chromatin Database (ChromDB), genes present on the RT-qPCR platform (Platform), differentially expressed (DE), and differentially alternatively spliced (DAS) genes, based on published data (Calixto et al., 2018). A gene was considered DE or DAS if it was differentially expressed or differentially spliced in at least one time point measured. **(B)** Significance of the overlap and representation factor for different pairwise comparisons. For details see **Supplemental Table 5**.

Although we did not observe changes in H3K27me3 occupancy at early cold-inducible genes after cold exposure, we wondered whether Pc-G, Pc-G associated and Pc-G antagonist genes and other chromatin genes are subject to transcriptional and/or post-transcriptional regulation upon cold-exposure. We analyzed this in two ways: first, we generated a RT-qPCR platform permitting expression analyses of Pc-G, Pc-G associated and Pc-G antagonist genes, histone and histone variant genes, and DNA methyltransferases and demethylases (in total 135 genes, see Methods and below). In addition, we extracted chromatin genes from the ChromDB (Gendler et al., 2008) and analyzed their transcriptional regulation and alternative splicing using a published dataset (Calixto et al., 2018). While chromatin regulatory genes were not significantly enriched among the genes differentially expressed in the cold (DE genes), they were highly enriched among the differentially alternatively spliced genes (DAS genes) (**Figure 3**). Only 14 out of 511 genes from ChromDB were both differentially expressed and spliced in the cold.

Nevertheless, interesting regulation patterns of several gene families were identified. Among the transcriptionally regulated genes were six histones and histone variants (*HTR2*, *HTR6*, *HTR11*; *HTA6*, *HTA12*; *H1.3*) and all three paralogues of the DNA demethylase *DEMETER*, *DML1*/*ROS1*, *DML2*, and *DML3* (**Supplemental Table 5**). Genes involved in vernalization were not differentially expressed in response to cold, however, several vernalization regulators showed alternative splicing events (7 out of 15 genes) (**Table 2**). These include *SR45*, *EMF2*, *VRN2*, *VRN5*, *VEL1*, *SWN*, and *HSL1*. For most genes, alternative splicing resulted in repression or induction of alternative variants which encode slightly altered proteins, e.g. for VRN2 a cold-induced alternative transcript translates into a protein with an addition of the amino acids QL at position 304 which is within the highly conserved VEFS domain (**Supplemental Figure 2**). Interestingly, among the genes that were both differentially expressed and spliced was the H3K27me3 demethylase *JMJ30*. Its close paralogue, *JMJ32* was also differentially expressed but not alternatively spliced. Overall, our bioinformatic analyses detected a widespread differential expression and splicing of chromatin regulators. Particularly, cold-induced alternative splicing of vernalization regulators is interesting and consistent with previously reported post-transcriptional regulation of *VRN2*, *FIE*, *CLF*, and *SWN* (Wood et al., 2006). Whether the proteins generated by alternative splicing exhibit different functional properties or have different interaction partners remains to be discovered.

**Table 2:** Summary of the regulation of genes involved/related to vernalization.

Gene	Name	ChromDB	RT-qPCR platform	Cold regulation on the platform	Cold regulation in Calixto et al., 2018
AT1G16610	SR45	No	No		Alternative splicing
AT2G23380	CLF	Yes	Yes	Upregulated after cold acclimation and after 2 h Deacc	None
AT2G30470	VAL1/HSI2	No	Yes	Upregulated after cold acclimation	None
AT2G45640	AtSAP18	Yes	No		None
AT3G18990	VRN1	Yes	Yes	Upregulated after cold acclimation	None
AT3G20740	FIE	Yes	Yes	Upregulated after cold acclimation	None
AT3G24440	VRN5	Yes	Yes	Downregulated after 12 h Deacc	Alternative splicing
AT4G02020	SWN	Yes	Yes	Downregulated after cold acclimation	Alternative splicing
AT4G16845	VRN2	Yes	Yes	None	Alternative splicing
AT4G30200	VEL1/VIL2	Yes	Yes	None	Alternative splicing
AT4G32010	HSL1	No	Yes	None	Alternative splicing
AT4G39680	ACINUS	No	No		None
AT5G17690	LHP1	Yes	No		None
AT5G51230	EMF2	Yes	Yes	None	Alternative splicing
AT5G57380	VIN3	Yes	Yes	None	None

Columns indicate the nomenclature of each gene, its presence in the Chromatin Database (ChromDB), on the RT-qPCR platform, and whether its expression is affected by cold in the RT-qPCR platform or in previously published data (Calixto et al., 2018). Alternative transcripts and encoded proteins are displayed in **Supplemental Figure 2**.

### Expression of Genes Encoding Proteins Involved in Epigenetic Processes During Cold Acclimation and Deacclimation

To allow expression analyses of Pc-G, Trx-G, and associated genes, histone genes, and genes involved in DNA methylation under various conditions, we set up a RT-qPCR platform. The expression of the 144 selected genes was analyzed in samples from NA, ACC, and deacclimated (Deacc) plants after 2, 4, 6, 12, and 24 h of deacclimation. Due to extremely low expression levels throughout all samples, gene expression data for *JUMONJI* (*jmc*) domain*-containing protein 14* (*JMJ14*) (AT4G20400), *Maternal affect embryo arrest 27/*(*JMJ15*) (AT2G34880), *JMJ17* (At1g63490), *JMJ18* (AT1G30810), *JMJ26* (At1g11950), *VP1/ABI3-like 3* (*VAL3*) (AT4G21550), *Probably E3-ubiquitin protein ligase* (*DRIPH*) (AT3G23060), *Chromomethylase 1* (*CMT1*) (AT1G80740), and *male-gamete-specific histone H3* (*MGH3*) (AT1G19890) were not further considered, resulting in 135 investigated genes.

Expression changes of these genes during cold acclimation and subsequent deacclimation are shown in five heat maps compiled according to the function of the respective proteins in epigenetic regulation. The corresponding normalized 2^−ΔCt^ values are shown in **Supplemental Table 6**.

**Figure 4:**
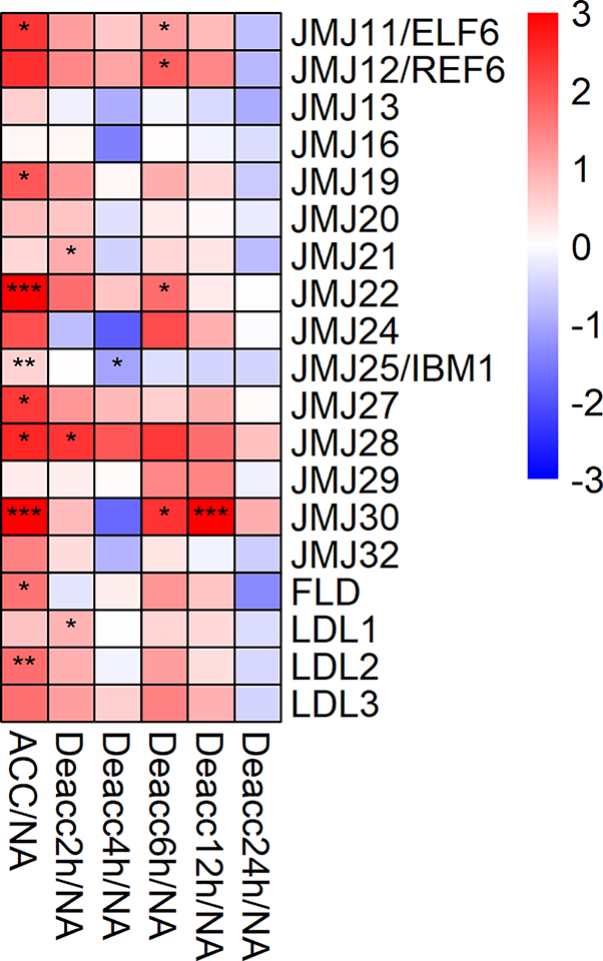
Expression changes of genes encoding JUMONJI-type and LSD1-type histone demethylases after cold acclimation (ACC) and after 2, 4, 6, 12, and 24 h of deacclimation (Deacc). Gene expression is presented as log2 fold change to non-acclimated conditions (NA) (**Supplemental Table 7**). The scale of log2 fold changes ranges from −3 (blue) to 3 (red) with a median of 0 (white). Significance levels are indicated relative to NA: ***P < 0.001; **P < 0.01; *P < 0.05.

The expression of 9 out of 19 genes encoding JUMONJI-type and lysine specific 1A-type (LSD1) histone demethylases was significantly upregulated after cold acclimation. Out of these nine genes, *JMJ11/ELF6*, *JMJ19*, *JMJ22*, *JMJ27*, *JMJ28*, and *JMJ30* showed the highest log_2_ fold change compared to NA (**Figure 4**). During deacclimation the expression of these genes decreased over time which is additionally illustrated in the comparison of gene expression levels at all deacclimation time points with the expression at ACC conditions (**Supplemental Figure 3**, **Supplemental Table 7**). However, most genes displayed a drop in expression after 4 h Deacc followed by a slight increase at 6 h Deacc. Almost all genes showed a significant downregulation of the expression relative to ACC after 24 h Deacc (**Supplemental Figure 3**) and no significant differences compared to NA conditions.

**Figure 5:**
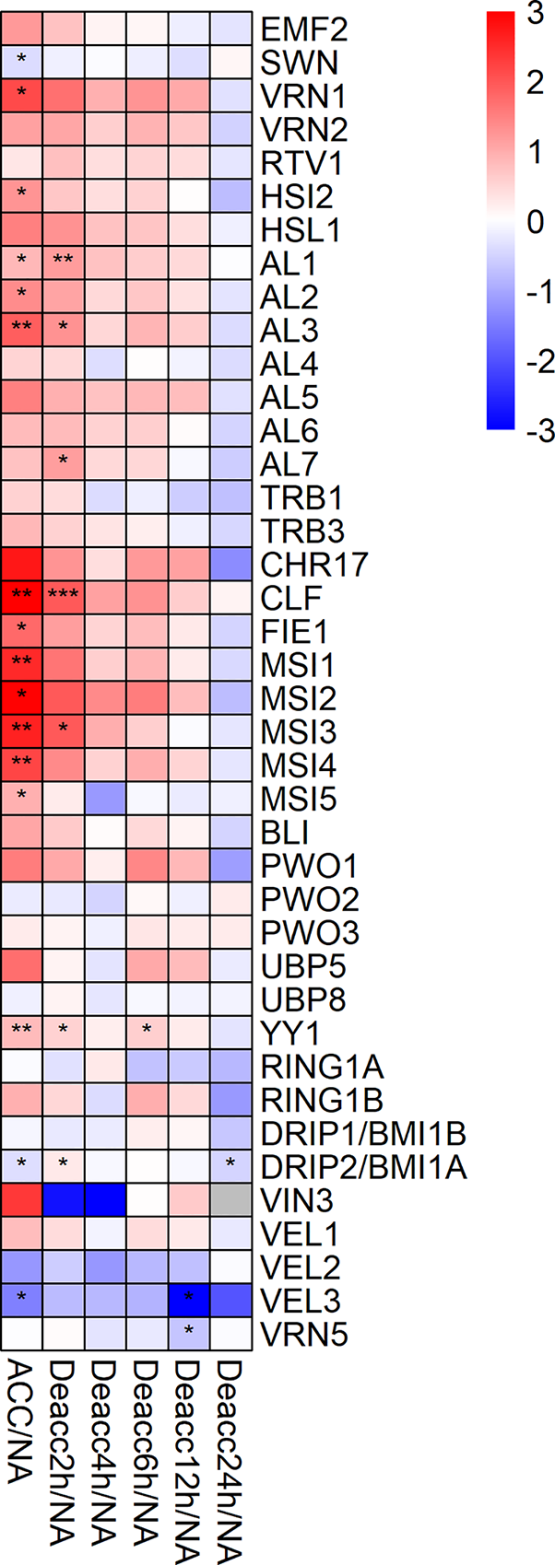
Expression changes of genes encoding proteins of the polycomb group (Pc-G) family after cold acclimation (ACC) and after 2, 4, 6, 12, and 24 h of deacclimation (Deacc). Gene expression is presented as log2 fold change to non-acclimated conditions (NA) (**Supplemental Table 7**). The scale of log2 fold changes ranges from −3 (blue) to 3 (red) with a median of 0 (white). Significance levels are indicated relative to NA: ***P < 0.001; **P < 0.01; *P < 0.05 *.

Forty genes encoding members of the Pc-G related protein family were included in the expression analysis (**Figure 5**, **Supplemental Figure 4**). Similar to the JUMONJI-type and LSD1-type histone demethylase families, most Pc-G related genes displayed an upregulation during cold acclimation, with the highest upregulation for *CLF*, four *WD40 repeat containing proteins MSI1-4* and *VRN1*. Altogether, 13 genes encoding Pc-G related proteins were significantly upregulated, while *SWN*, *DRIP2/BMI1A*, and *VEL3* exhibited a downregulation at ACC. Most genes with a strong upregulation at ACC kept their higher expression levels over 12 h of deacclimation before they were significantly downregulated in comparison to ACC after 24 h (**Supplemental Figure 4**, **Supplemental Table 7**). After 6 h Deacc their expression was either increased transiently before returning to the initial NA expression levels or was continuously downregulated during the 24 h of deacclimation (**Figure 5**). Ten genes displayed a decrease in expression during deacclimation, which was significant in comparison to ACC over several time points, including *AL3*, *CLF*, *FIE1*, *MSI1-MSI5*, *YY1*, and *VIN3* (**Supplemental Figure 4**). *VEL2* was the only gene with a significant upregulation in comparison to ACC after 24 h Deacc (**Supplemental Figure 4**). *DRIP2/BMI1A*, on the other hand, was the only gene of the Pc-G related protein family with a significantly reduced expression at 24 h Deacc compared to NA.

**Figure 6:**
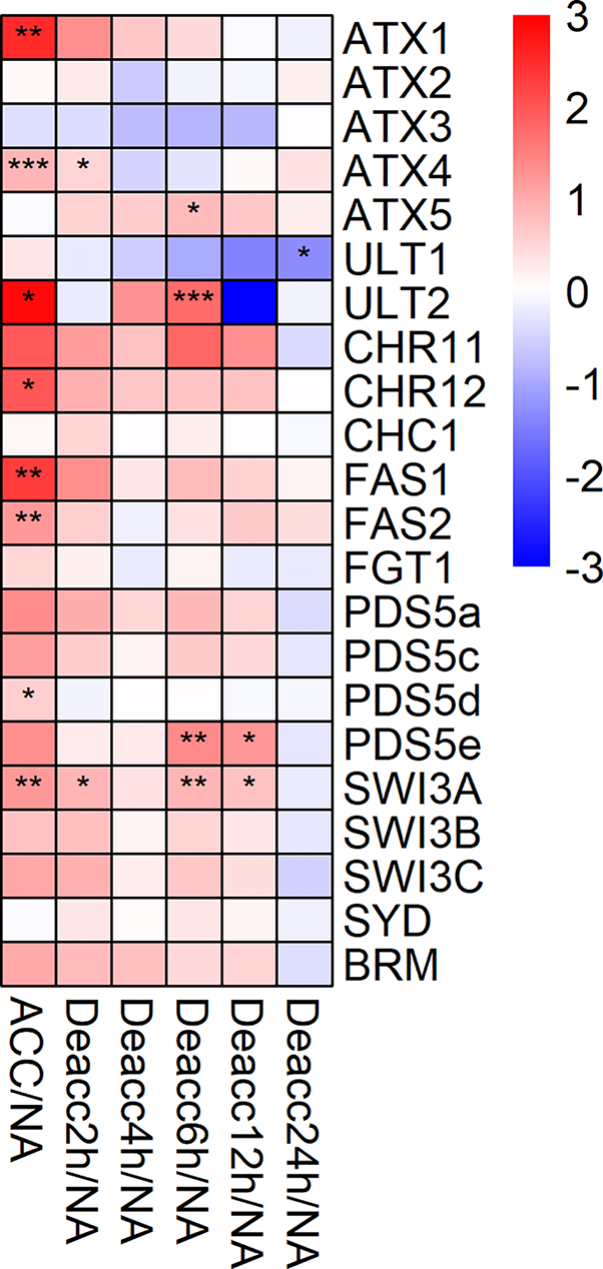
Expression changes of genes encoding proteins of the trithorax group (Trx-G) after cold acclimation (ACC) and after 2, 4, 6, 12, and 24 h of deacclimation (Deacc). Gene expression is presented as log2 fold change to non-acclimated conditions (NA) (**Supplemental Table 7**). The scale of log2 fold changes ranges from −3 (blue) to 3 (red) with a median of 0 (white). Significance levels are indicated relative to NA: ***P < 0.001; **P < 0.01; *P < 0.05 *.

The expression of 22 genes encoding Trithorax-group (Trx-G) related proteins and chromatin remodelers was also investigated (**Figure 6**, **Supplemental Table 7**). Eight of these genes were significantly induced during cold acclimation (*ATX1*, *ATX4*, *ULT2*, *CHR12*, *FAS1*, *FAS2*, *PDS5d*, and *SWI3A*). After 2, 6, and 12 h Deacc only two, four, and two of these genes, respectively, were still significantly induced with only *SWI3A* showing a stable significant induction over almost all time points. A significant transiently changed expression over two time points during deacclimation compared to NA was only evident for *PDS5e* at 6 h and 12 h. At 24 h Deacc expression changes caused by cold acclimation were mostly reversed and expression of all genes reached similar levels as under NA conditions, except for *ULT1*, which was significantly downregulated. *ATX5* displayed a transient upregulation in comparison to ACC conditions till 12 h Deacc (**Supplemental Figure 5**).

**Figure 7:**
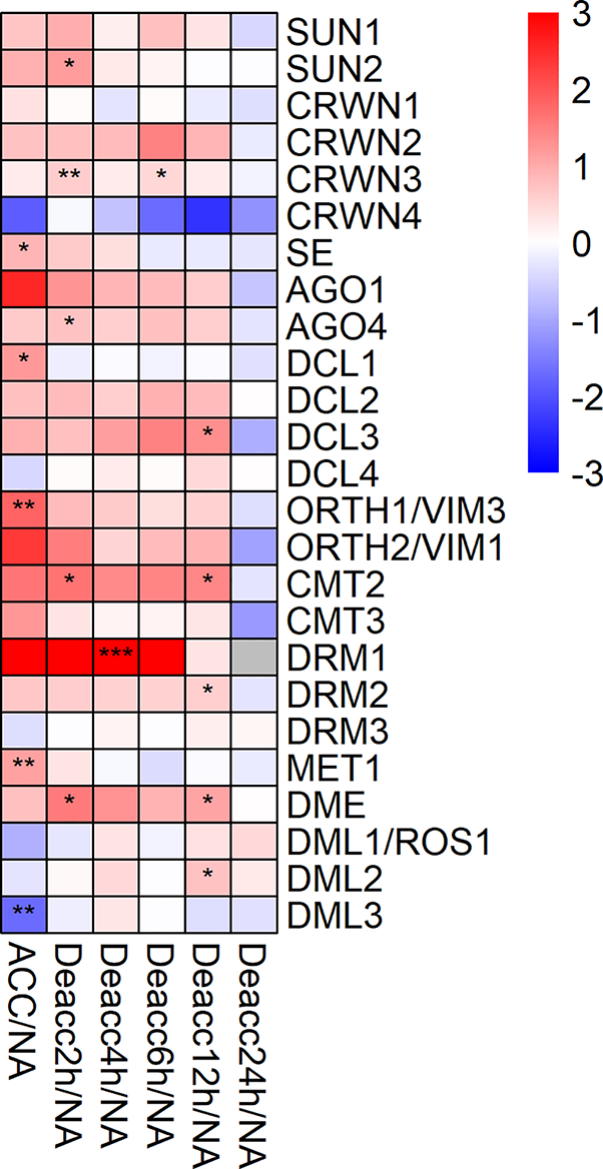
Expression changes of genes encoding proteins acting in chromosome-nuclear envelope (Chr-NE) interactions, RNA interference and methylation after cold acclimation (ACC) and after 2, 4, 6, 12, and 24 h of deacclimation (Deacc). Gene expression is presented as log2 fold change to non-acclimated conditions (NA) (**Supplemental Table 7**). The scale of log2 fold changes ranges from −3 (blue) to 3 (red) with a median of 0 (white). Significance levels are indicated relative to NA: ***P < 0.001; **P < 0.01; *P < 0.05.

Further, the expression of 25 genes encoding proteins acting in chromosome-nuclear envelope (Chr-NE) interactions, RNAi and DNA methylation was measured (**Figure 7**, **Supplemental Figure 6**). Five genes of this group were significantly differentially expressed at ACC compared to NA (**Figure 7**), *SE*, *DCL1*, *ORTH1/VIM3*, *MET1*, and *DML3*. Only *DML3* was significantly reduced in its expression under ACC compared to NA conditions. For seven genes of this group expression decreased significantly at different time points of deacclimation compared to ACC, *SUN2*, *SE*, *DCL1*, *ORTH1/VIM3*, *DRM3*, *MET1*, and *DML3* (**Supplemental Figure 6**, **Supplemental Table 7**). In contrast, especially *CMT2*, *DRM1*, and *DME* displayed a stable upregulation until 12 h Deacc or 6 h Deacc (**Figure 7**). *DML3* was highly induced at almost all time points of deacclimation compared to ACC and was together with *DRM3* and *DML1/ROS1* still significantly upregulated compared to ACC at 24 h Deacc (**Supplemental Figure 6**).

**Figure 8:**
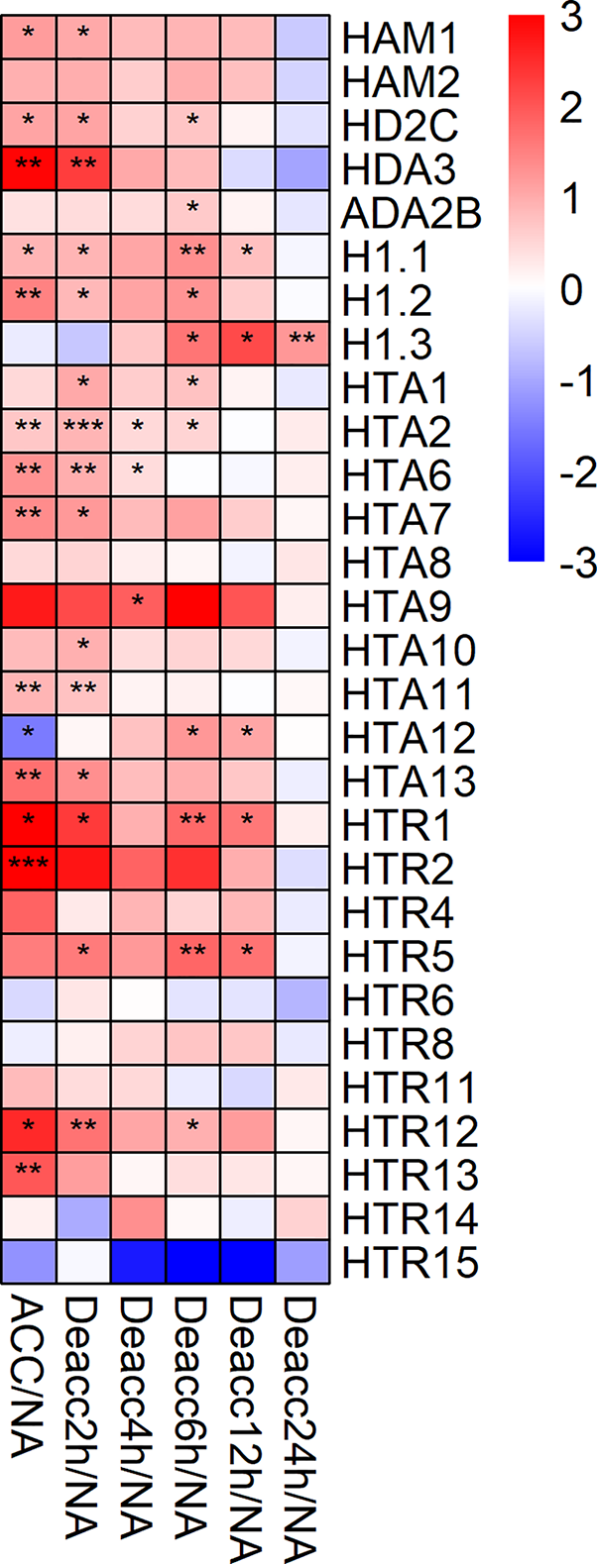
Expression changes of genes encoding histone acetyltransferases (HAC), deacetylases (HDAC), or histone variants after cold acclimation (ACC) and after 2, 4, 6, 12, and 24 h of deacclimation (Deacc). Gene expression is presented as log2 fold change to non-acclimated conditions (NA) (**Supplemental Table 7**). The scale of log2 fold changes ranges from −3 (blue) to 3 (red) with a median of 0 (white). Significance levels are indicated relative to NA: ***P < 0.001; **P < 0.01; *P < 0.05 *.

Lastly, changes in expression levels of 29 genes that encode HAC, histone deacetylases (HDAC), or histone variants were investigated (**Figure 8**, **Supplemental Figure 7**). Fourteen of the selected genes were significantly upregulated after 3 days of cold acclimation, including *HDA3*, *HTR1* and *HTR2*, *HTR12*, and *HTR13*, which showed the highest induction (**Supplemental Table 7**). Strikingly, this upregulation was still present after 2 h Deacc for 12 of these genes and became significant for three additional ones, *HTA1*, *HTA10*, and *HTR5*. About half of the genes displayed upregulated expression in comparison to NA over 6 h of deacclimation before they were downregulated after 24 h. Especially for *H1.1*, *HTR1*, and *HTR5*, a more stable upregulation was observed which was still significant after 12 h Deacc. These genes displayed an increase in expression after cold acclimation, followed by a slight decrease up to 4 h of deacclimation compared to NA conditions. From 4 to 12 h Deacc, expression increased again before returning to the NA level after 24 h, similarly to the pattern in most JUMONJI family genes.

Interestingly, *HTA12* was upregulated at later time points of deacclimation (6 and 12 h Deacc) after exhibiting the largest decrease in expression after cold acclimation in comparison to NA. Consistently, this gene was significantly induced compared to ACC along the whole deacclimation time course (**Supplemental Figure 7**). Lastly, *H1.3* was the only investigated gene that displayed a decrease after cold acclimation and an increase throughout the 24 h Deacc (**Supplemental Figure 7**, **Supplemental Table 7**).

## Discussion

### H3K27me3 Preferentially Targets Early Cold Inducible and Late Repressed Genes

Chromatin and chromatin modifications contribute to the regulation of stress-regulated genes at various layers: (1) the repression of stress-inducible genes in non-stress conditions (by repressive chromatin), (2) the activation or repression of genes immediately after stress exposure (by the acquisition of active or repressive chromatin, respectively), (3) the sustained activation or repression in non-stress conditions after exposure to the stress, and (4) the transcriptional memory of a stress (in non-stress conditions), permitting primed gene regulation when exposed again to the stress (Friedrich et al., 2019). It is therefore conceivable that chromatin genes and the activity of their gene products are regulated at various layers during different phases of stress exposure and relief. Pc-G-mediated H3K27me3 is one of the key repressive chromatin modifications and targets thousands of genes in non-stress conditions, which are developmental regulators, tissue-specifically regulated genes and stress-responsive genes (Lafos et al., 2011). By bioinformatics comparison of H3K27me3 target genes and a detailed kinetic analysis of cold-regulated genes, we revealed an enrichment of H3K27me3 target genes among the early inducible genes. As the early inducible genes are required to trigger a cascade of gene regulatory networks to permit cold acclimation, it is likely particularly important to control their tight repression in non-stress conditions. Induction of H3K27me3-silenced genes may occur *via* different mechanisms: the enzymatic removal by histone demethylases, the addition of active modifications leading to a bivalent chromatin state, the exchange of histones by histone variants and the removal or sliding of nucleosomes by chromatin remodeling complexes. Interestingly, several genes encoding proteins potentially regulating dynamic changes in H3K27me3 are induced by cold, including the H3K27me3 demethylase *JMJ30* (Gan et al., 2014), the Trx-G proteins *ATX1* (a H3K4 methyltransferase) and *ULT1* (Alvarez-Venegas et al., 2003; Carles and Fletcher, 2009), and diverse histone variants. At least for the analyzed cold-inducible H3K27me3 genes *GRXS4*, *TCF1*, *ULT1*, and *WRKY48*, we did not detect changes in H3K27me3 occupancy upon cold exposure, thus it is likely that these genes are activated by acquisition of activating marks or show only depletion of H3K27me3 at specific nucleosomes or in a tissue-specific manner. As activation of *COR15A* is associated with a reduction in nucleosome occupancy (which we did not detect for other genes), there are likely different, gene specific mechanisms at work. Our analyses also revealed that H3K27me3 target genes (identified in warm conditions) are overrepresented among the late repressed genes in the cold (**Figure 1**). We did not uncover changes in H3K27me3 occupancy upon cold exposure, at least for *PRR1*, suggesting that the mode of (further) repression is not accompanied by increased H3K27me3. Also here, H3K27me3 may be increased at specific nucleosomes, which is observed for the H3K27me3 target gene *FLOWERING LOCUS C* (*FLC*) which carries H3K27me3 in the warmth but a higher coverage/spreading of H3K27me3 upon cold exposure/vernalization (Schubert et al., 2006; De Lucia et al., 2008). The late repressed H3K27me3 target genes may be marked for long-term repression in the cold, similar to *FLC*.

### Expression Analysis of Genes Mediating Epigenetic Changes

In general, our analysis indicated that cold acclimation had a strong influence on the expression of the selected epigenetics-related genes. This is in agreement with the finding that cold stress enhanced the accessibility of chromatin and bivalent histone modifications of active genes in potato (Zeng et al., 2019). After 24 h of deacclimation, expression of the investigated genes had largely returned to the NA status. This is in agreement with our earlier data on the expression of genes encoding transcription factors, also investigated by RT-qPCR, and global gene expression, investigated by microarray hybridization (Pagter et al., 2017).

### Genes Encoding JUMONJI-Type and LSD1 Histone Demethylases are Upregulated During Cold Acclimation

JUMONJI-type and LSD1-type enzymes are histone demethylases. For several years histone methylation was thought to be irreversible until the discovery of human LSD1 (Mosammaparast and Shi, 2010). Although members of both enzyme families target histone methylation, they have different structures as well as targets. The JUMONJI family is defined by a JUMONJI C (JmjC) domain consisting of two histidines and one glutamate residue to chelate the catalytic iron, which is essential for its function (Mosammaparast and Shi, 2010). JmjC proteins are able to demethylate tri-methylated lysines, including those on H3K9, H3K27 and H3K36 (Mosammaparast and Shi, 2010; Gan et al., 2014). JmjC-domain proteins further reverse trimethylated H3K4 to its mono- or dimethylated forms (Iwase et al., 2007). LSD1 histone demethylases contain a SWIRM domain with an amine oxidase domain containing a substrate binding and an FAD-binding part and are only able to demethylate mono- and dimethylated lysines, such as H3K4me1/2 (Stavropoulos et al., 2006; Yang et al., 2006).

It is interesting to note that several members of both families of demethylases displayed an upregulation during cold acclimation before decreasing until 4 h Deacc followed by an increase until 12 h Deacc before returning to the initial NA gene expression levels after 24 h Deacc. This suggests that both demethylase classes are required during cold acclimation and deacclimation. The elevated expression of members of both gene families coincides with previous results, where a reduction in H3K9 methylation during short-term cold stress was described (Hu et al., 2012). Research has further shown that *JUMONJI* and *LSD1* genes are linked to regulation of developmental transitions in *A. thaliana*. For example, histone demethylation of the *FLC* gene by *JMJ30* and *JMJ32* controls flowering at warm temperatures (Yang et al., 2010; Gan et al., 2014). Further, *JUMONJI* genes are upregulated under drought stress in peanut (Govind et al., 2009; Shen et al., 2014), in agreement with the upregulation found in ACC samples in this experiment. Lastly, JUMONJI proteins have been associated with changes in the circadian clock (Jones et al., 2010; Lu et al., 2011a). As the samples analyzed here were harvested throughout a time period of 24 h during deacclimation, circadian regulation could have contributed to the expression changes of e.g. *JMJ30* (Lu et al., 2011b). Additional experiments will be necessary to clarify this contribution. Nevertheless, results for ACC and 24 h Deacc plants are not affected, as these samples were collected at the same time of day as the NA samples. Therefore we conclude that the majority of the investigated histone demethylases of the JUMONJI-type and LSD1 family are upregulated during cold acclimation.

### Pc-G Proteins are Involved in Epigenetic Changes During Cold Acclimation and Deacclimation

The Pc-G gene family was discovered in *Drosophila*, where its members encode proteins able to repress the *HOX* genes (Lewis, 1978). The Pc-G gene family encodes a diverse set of proteins with a variety of molecular activities (Sauvageau and Sauvageau, 2010; Del Prete et al., 2015). Polycomb proteins act as multiprotein complexes, Polycomb Repressive Complex 1 (PRC1) and PRC2 in plants and PRC3 in humans (Schwartz and Pirrotta, 2007; Kleinmanns and Schubert, 2014). The PRC1 represses genes through mono-ubiquitination of histone H2A and chromatin remodeling (Kleinmanns and Schubert, 2014). Several *PRC1* genes were investigated, such as *RING1a*, *RING1b* as well as *EMBRYONIC FLOWER 1 (EMF1)*. PRC2 mediates the trimethylation of H3K27 (H3K27me3), which results in the repression of transcription through changes in chromatin organization (Kleinmanns and Schubert, 2014; Del Prete et al., 2015).

Most genes encoding proteins of the Pc-G displayed an upregulation during cold acclimation followed by either a relatively stable expression or decreased expression during deacclimation. Polycomb proteins have been linked to abiotic and biotic stresses in *A. thaliana*. WD-40 repeat containing protein MSI1 negatively regulates drought-stress responses in *A. thaliana* and a knockout of this gene confers increased drought tolerance (Alexandre et al., 2009).

Furthermore, H3K27me3 and/or H3 levels decrease at the cold-inducible genes *COR15A* and *GOLS3* during cold stress. Upon transfer to ambient temperatures low H3K27me3 were maintained while *COR15A* and *GOLS3* were repressed again. Thus, H3K27me3 is not sufficient to inhibit transcription, but the gene activation rather leads to H3K27me3/H3 removal (Kwon et al., 2009).

Further research on the regulation of cold-responsive genes by proteins of the Pc-G showed that *EMF1* and *EMF2* repress several cold-regulated genes such as *COR15A* and *CBF1* (Kim et al., 2010b). Similarly, MSI4/FVE was identified in a screen for repressors of *COR15A* and loss of FVE leads to higher freezing tolerance in ACC plants (Kim et al., 2004).

As many cold-inducible genes carry the PRC2 mark H3K27me3 in the warmth, these genetic analyses are consistent with an important function for Pc-G proteins in prevention of precocious expression of cold-inducible genes. Although we found an increase in the expression of most *PRC2* genes during cold acclimation, PRC2 occupancy analyses during cold acclimation will be required to reveal their presence on the cold-inducible genes. Results collected in this work suggest that a higher expression of genes encoding specific PRC2 subunits, such as *WD-40 repeat-containing proteins* (*MSI1-MSI5*), at ACC conditions might have led to an increase or redistribution of H3K27me3, resulting together with other changes in the increased freezing tolerance of *A. thaliana*. Supporting this hypothesis, induction of Pc-G genes during cold acclimation has also been reported previously. *Brassica oleracea* displayed induced expression of alfin-like transcription factors, which are interactors of PRC1, during 24 h at 4°C (Kayum et al., 2016) which is consistent with the observed induction of alfin-like genes in our study.

PRC1 and PRC2 proteins are central regulators of vernalization (Song et al., 2012). The only reported Pc-G protein induced by cold is *VERNALIZATION INSENSITIVE 3 (VIN3)* which is only induced after prolonged cold (at least 10 d) (Sung and Amasino, 2004; Kim et al., 2010a). We did not observe this induction as plants only experienced a 3-day cold period, however, a cold induction of *VRN1*, *CLF*, *VAL1*, and *FIE* was shown in this work. In addition, several vernalization-related genes were regulated by alternative splicing in the cold, including the PRC2 genes *SWN*, *VRN2*, and *EMF2* (**Table 2**). Importantly, all of the alternatively spliced transcripts result in proteins with modified amino acid sequence. Whether these variants have a different function, stability or interaction partners remains to be determined. Wood et al. (2006) revealed that Pc-G proteins are also regulated at the post-translational level as VRN2, CLF, FIE, and SWN showed higher protein abundance after prolonged cold, while no changes in steady-state mRNA levels are detected. In conclusion, our and previous work suggest that Pc-G genes are regulated at the transcriptional, post-transcriptional (alternative splicing), and post-translational level. As the PRC2-mediated H3K27me3 appears to be a major mark repressing cold-regulated genes, tight regulation of PRC2 in the cold is important. Whether PRC2 regulation relates to cold acclimation and chilling/freezing tolerance in addition to vernalization remains to be determined.

### The Stress-Responsive *ATX1* Gene of the Trx Group is Cold Induced

Trx-G proteins act as antagonists to Pc-G and activate Pc-G target gene transcription by depositing H3K4me3 (Del Prete et al., 2015). Consequently, the activity of these genes must be finely tuned by opposing actions of these protein complexes (Del Prete et al., 2015). Further Trx-G proteins can also trimethylate H3K36 to activate transcription of target genes and act as an ATP-dependent chromatin remodeling complex, such as proteins containing a SWITCH or Brahma domain (Schuettengruber et al., 2011; Del Prete et al., 2015). Trx-G proteins such as ATX1 (an H3K4 methyltransferase), which plays a role in drought tolerance, have been linked to abiotic and biotic stresses in *A. thaliana* (Ding et al., 2011). This gene, together with *ATX4*, was also highly induced after cold acclimation. A loss of *ATX1* expression results in decreased germination rates, larger stomatal apertures and thus higher transpiration rates, as well as lower drought tolerance (Ding et al., 2011). Further, binding of ATX1 to the gene *9-cis-epoxycarotenoid dioxygenase 3* (*NCED3*), encoding a protein catalyzing the limiting step in ABA synthesis, was observed, and a loss of ATX1 resulted in decreased *NCED3* levels under drought stress (Ding et al., 2011). ATX1 has also been linked to the regulation of the salicylic acid and jasmonic acid pathways *via* WRKY70, which is a regulator of the plants defense pathway (Alvarez-Venegas et al., 2007).

The gene *ATP-dependent helicase BRAHMA* (*BRM*), encoding an ATP-dependent chromatin remodeling complex, displayed opposite effects to *ATX1* under drought stress, resulting in increased drought tolerance when the gene was non-functional, through repression of *ABA INSENSITIVE5* (Han et al., 2012). While *BRM* expression is not altered by cold, its paralog *CHR12* is induced after cold acclimation. CHR12 is required to arrest growth after the plant is exposed to a stress (drought, heat, salt) (Mlynarova et al., 2007). Thus, its induction may be directly linking growth and stress responses. Three other genes that are highly induced during cold acclimation, *FAS1*, *FAS2*, and *MSI1* encode proteins which form subunits of CHROMATIN ASSEMBLY FACTOR 1 (CAF-1) and are involved in maintaining the cellular organization of the shoot apical meristem (Kaya et al., 2001). *SWI3A* was the only gene of this group which was highly expressed during the first 12 h of deacclimation. It plays an essential role for plant growth and development (Zhou et al., 2003). These results show that there is a finely tuned regulation of several Trx-G genes and chromatin remodelers involved in abiotic stress response and stress release. Regulatory functions of Trx-G during cold stress have not been reported yet. Nevertheless, the involvement of many of these genes in stress responses in plants suggests a possible participation. Furthermore results of this work suggest that genes encoding Trx-G proteins play a role in cold acclimation in *A. thaliana*. Some proteins of Trx-G may also be involved in deacclimation, but further experiments would be required to investigate this.

### DNA Methylation May Play a Role in Deacclimation

One group of DNA methylases adds a methyl group onto cytosine residues in higher eukaryotes and have been proposed to control gene expression in plants during development and regulate transposable elements and heterochromatin (Finnegan et al., 1996; Finnegan et al., 1998; Pavlopoulou and Kossida, 2007). Chromomethylases (CMT) are plant-specific and have been linked to symmetric and asymmetric methylation of DNA (Bartee et al., 2001). Changes in the levels of DNA methylation are known to occur during abiotic stresses; however their exact functions and effects are still unclear. For example, it has been observed that a hypermethylation of DNA occurs during salt stress in wheat (Peng and Zhang, 2009). Some of the investigated genes might be involved in deacclimation, e.g. *CMT2* displayed a continued increase in expression up to 12 h Deacc. Furthermore, DNA demethylases such as DEMETER have been previously linked to plant stress responses and *DME* also showed increased expression during deacclimation. In addition, deletion of three DNA demethylases (*DML1*, *DML2*, *DML3*) resulted in increased susceptibility to fungal pathogens and therefore a participation in the biotic stress response of plants was proposed (Le et al., 2014). Interestingly, *DML3* was significantly increased in comparison to ACC at all deacclimation time points and three out of four demethylases (*DML1*, *DML2*, *DML3*) were still upregulated at 12 and/or 24 h Deacc, suggesting that deacclimation is accompanied by a resetting of DNA methylation.

RNAi-related proteins are commonly found in the nucleus and cytoplasm and are well-known to act in post-transcriptional gene silencing in the cytoplasm (Castel and Martienssen, 2013). Dicer and DICER-LIKE (DCL) proteins are key regulators of small RNA biogenesis (RNAi). Only *DCL1* was induced by cold acclimation whereas *DCL3* was significantly upregulated only after 12 h Deacc. Expression analyses on *DCL* genes in rice showed differential responses comparing drought, cold, and salt stress (Liu et al., 2009). However, the cold response of *DCL* genes in rice differed compared to *Arabidopsis*, which may be due to the fact that rice, in contrast to *Arabidopsis*, is a chilling sensitive plants.

### Histone Variants Respond Differentially and Strongly to Cold Acclimation and Deacclimation

Histone acetylation can occur on 26 potential lysine residues in a nucleosome (Lusser et al., 2001) and is a reversible process. Acetylation can alter the surface of nucleosomes and destabilize it to enhance binding of proteins to transcribed regions (Berger, 2007). Our results indicate an induction of both HDACs and HAC during cold acclimation and deacclimation. HDACs have been previously linked to responses to drought and salt stress, but not cold, in young rice seedlings (Hu et al., 2009). In *Zea mays* HDACs were induced during cold treatment, resulting in deacetylation of histone subunits H3 and H4. In addition, a direct activation of *ZmDREB1* expression by ZmHDACs was suggested (Hu et al., 2011). In *Arabidopsis*, *HDA6* is involved in cold acclimation through the regulation of cold-responsive genes (To et al., 2011; Kim et al., 2012). Similarly, the expression of *HDA3* was highly induced during cold acclimation, and after 2 h Deacc, whereas *HDA6* was not investigated. A similar expression pattern as for *HDA3* was observed for *HD2C*. HD2A, HD2C, and HD2D interact with HDA6 and HDA19 in multiprotein complexes (Luo et al., 2017), and HD2C also interacts with BRAHMA, a chromatin remodeler involved in negative regulation of heat-responsive genes (Buszewicz et al., 2016). HD2C and the WD-40-repeat containing protein HOS15 interact before binding to the promoters of the cold-responsive genes *COR15A* and *COR47*. The cold induction of HOS15-mediated chromatin changes promotes HD2C degradation and is correlated with higher histone acetylation levels on the chromatin of *COR* genes. Additionally, HOS15 recruits CBF transcription factors to *COR* gene promoters (Park et al., 2018). The reported HD2C degradation seems to be contradictory to a higher *HD2C* gene expression under cold conditions, but an analysis of proteins levels will be necessary to compare these studies. Furthermore, the HAC Gcn5 (not investigated in this work) interacts with transcriptional adapter ADA2B, a transcriptional activator of HACs, and T-DNA insertions of *GCN5* lower the induction of *COR* genes during cold acclimation (Stockinger et al., 2001; Vlachonasios et al., 2003). The *ADA2B* gene was also induced during 6 h Deacc in this work, pointing to a possible activation of HACs.

Additionally, the expression of several genes encoding histone variants has been investigated. Variants of H2A and H3 are incorporated into the chromatin during the interphase of the cell cycle to confer unique properties to the nucleosome (Deal and Henikoff, 2011). Histone variants of the canonical H2A (*HTA2*, *HTA10*, *HTA13*), H2A.Z (*HTA8*, *HTA9*, and *HTA11*) and H2A.W (*HTA6*, *HTA7*, *HTA12*) were included in the analysis, as well as the histone H1 variants *H1.1*, *H1.2*, *H1.3*, and *HTR1* to *HTR15* from the histone H3 (Jiang and Berger, 2017). The expression of most genes encoding histone variants was induced during cold acclimation and stayed upregulated during deacclimation. Studies on temperature stress have discovered that H2A.Z variants are regulated by a mild increase in ambient temperatures (Kumar and Wigge, 2010). A recent model stresses the importance of the H2A.Z status for the transcriptional regulation (Asensi-Fabado et al., 2017). Under cold conditions, H2A.Z deposition is increased resulting in higher plant sensitivity to changes in temperature.

An interesting expression pattern was detected for histone variant *H1.3*, which was not changed during cold acclimation, but was the only gene displaying a significant and stable upregulation after 6 to 24 h Deacc, indicating that *H1.3* is not induced by cold, but specifically by deacclimation. *H1.3* was also found to be upregulated after 24 h Deacc compared to ACC conditions in a comparison of three publicly available data sets using microarray and RNA-Seq data and was considered to be part of a core set of 25 common upregulated genes during deacclimation (Vyse et al., 2019). *H1.3* is drought stress-induced in *A. thaliana*, and also responds to ABA treatment (Ascenzi and Gantt, 1997). A model for the action of histone 1 variants suggests that the small and mobile histone H1.3 replaces the canonical histone variants (H1.1 and H1.2) under stress conditions causing hypermethylation, but the influence of this process on transcriptional regulation and physiological responses is not clear yet (Asensi-Fabado et al., 2017). A similar pattern as for *H1.3*, but without induction at 24 h Deacc was found for *HTA12*. An induction of *H1.3* and *HTA12* during later deacclimation time points indicates a possible role of these genes in memorizing a previous stress event.

Overall, this study shows that many chromatin genes are dynamically transcriptionally and post-transcriptionally regulated during the plant cold response and deacclimation. Further work, especially genetic analyses, will be needed to investigate the function of these genes for both processes in more detail. In addition, the modifications set or removed by chromatin enzymes will require a detailed analysis. As hundreds of genes which are stably repressed in non-stress conditions and are targeted by H3K27me3 are activated within minutes after cold exposure, resetting of epigenetic information can be studied during the cold stress response. How this resetting results in memory of stress and/or induces vernalization, is an exciting question to address in the future.

## Data Availability Statement

All datasets generated for this study are included in the article/**Supplementary Material**.

## Author Contributions

KV, DS, and EZ designed the qRT-PCR platform. KV performed primer design, qRT-PCR experiments, and data analysis with input from EZ and DH. MP performed deacclimation experiments and prepared RNA. LF performed ChIP-qPCR and data analyses. LF and MR performed bioinformatic analyses. KV, DS, DH, and EZ wrote the manuscript.

## Funding

This work was supported through funds for project A3 to DH and C7 to DS from the Collaborative Research Center 973 funded by the German Research Foundation (DFG, SFB 973). We gratefully acknowledge support to MP through the People Program (Marie‐Curie Actions) of the European Union's Seventh Framework Programme (FP7 People: Marie‐Curie Actions FP7‐MC‐IEF) under REA grant agreement 328,713. We acknowledge support by the German Research Foundation and the Open Access Publication Fund of the Freie Universität Berlin.

## Conflict of Interest

The authors declare that the research was conducted in the absence of any commercial or financial relationships that could be construed as a potential conflict of interest.
