# Supplementary material for: Transcriptional and Post-Transcriptional Regulation and Transcriptional Memory of Chromatin Regulators in Response to Low Temperature
Source: Front Plant Sci. 2020 Feb 7;11:39. doi: 10.3389/fpls.2020.00039 (PMC7020257; doi:10.3389/fpls.2020.00039)
Supplement: Supplementary file 1 [file Image_1.pdf]

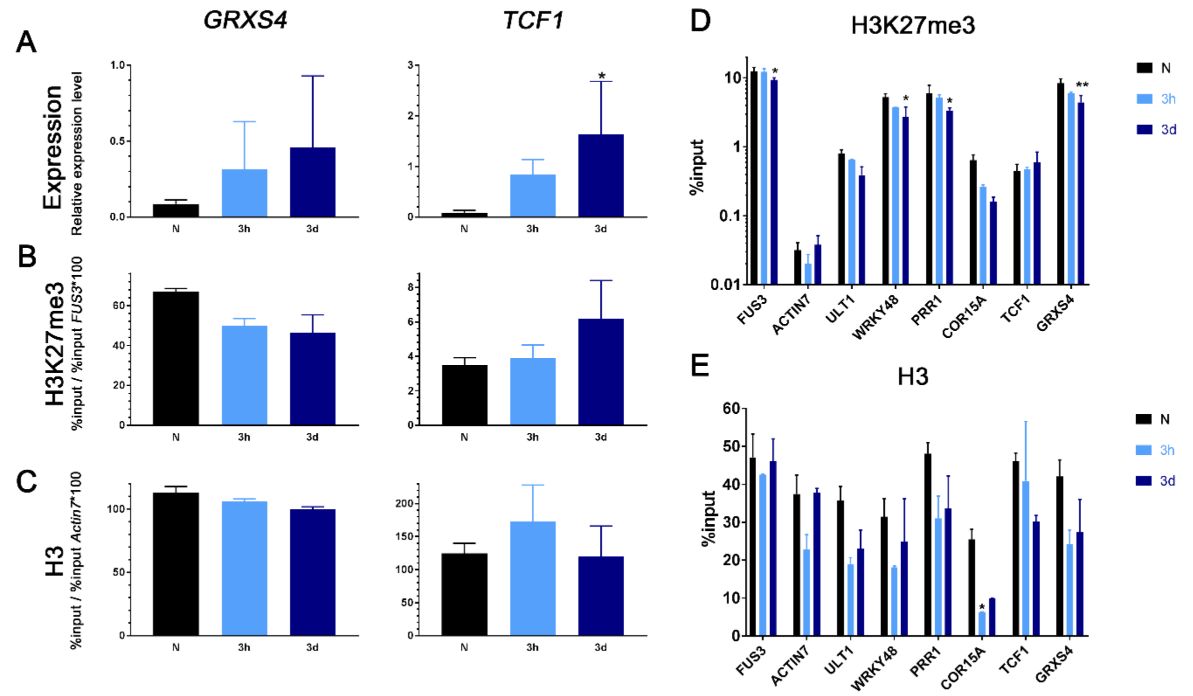

Supplemental Figure 1: Gene expression changes, H3K27me3 and H3 levels of early up-regulated (*GRXS4*, *TCF1*) genes as well in plants exposed to cold. (**A**) RNA was isolated from 3 weeks old seedlings grown at ambient temperature (N) and exposed to 4°C for 3h or 3d. Transcript levels for *GRXS4* and *TCF1* were measured by reverse transcription and real-time quantitative PCR. *ACTIN2*, *PDF* and AT4G34270 were used as internal control. Error bars indicate ± s.e.m., n = 3 biological replicates. Test for significance by one-way ANOVA followed by a Dunnett's multiple comparison test. Significance levels are indicated relative to N: *, P < 0.05. ChIP-PCR analysis of H3K27me3 levels (**B**) and H3 levels (**C**). Chromatin was extracted from the same seedlings after cross-linking and precipitated using H3K27me3 and H3 antibodies respectively. The purified DNA was amplified by real-time quantitative PCR. Results are presented as %input * 100 / %input at the *FUS3* locus for H3K27me3 and at the *ACTIN7* locus for H3. For H3K27me3, *ACTIN7* was used as a negative control. (**D**) Levels of H3K27me3 on all investigated locus as a %input, log10 scale. (**E**) Levels of H3 on all investigated locus as %input. Error bars on (**B**), (**C**), (**D**) and (**E**) indicate ± s.e.m, n = 2 biological replicates. Test for significance by one-way ANOVA followed by a Dunnett's multiple comparison test. Significance levels are indicated relative to N: *, P < 0.05, **, P < 0.01. All primer sequences used for this experiment can be found in Suppl. Table 1.

Supplemental Figure 2: Changes in the alternative splicing of vernalization actors during cold exposure and alignments of the translated variants. The expression profiles were obtained using the webservice at https://wyguo.shinyapps.io/atrtd2_profile_app/ (Calixto et al., 2018; Zhang et al., 2017) and the alignments were made using the Needle algorithm using the translated transcripts extracted from AtRTD2. The alignments were plotted using the Sequence Manipulation Suite.

SR45 – AT1G16610

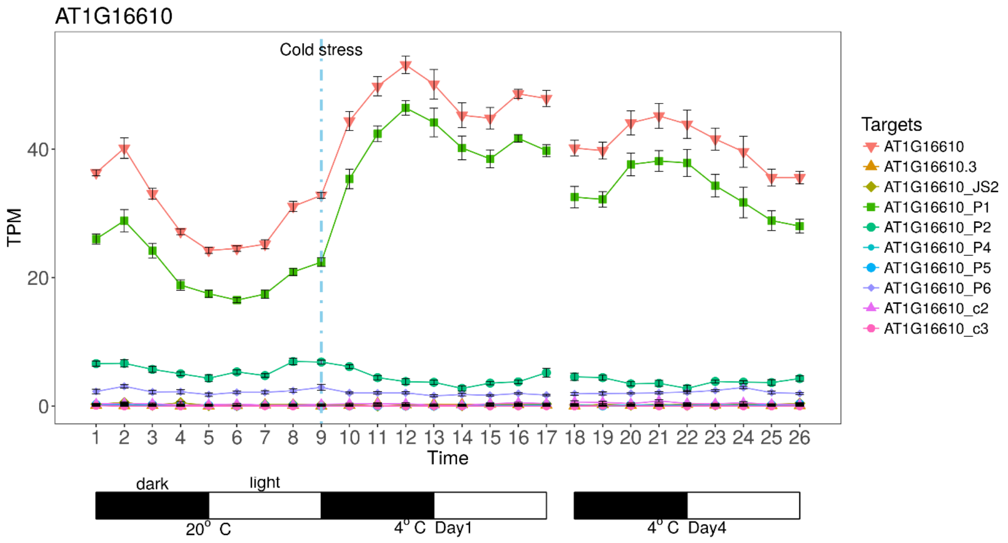


```
AT1G16610_P1   MAKPSRGRRSPSVSGSSSRSSSRSRSGSSPSRSISRSRSRSRSLSSSSSP   50
AT1G16610_P2   MAKPSRGRRSPSVSGSSSRSSSRSRSGSSPSRSISRSRSRSRSLSSSSSP   50

AT1G16610_P1   SRSVSSGSRSPPRRGKSPAGPARRGRSPPPPPSKGASSPSKKAVQESLVL   100
AT1G16610_P2   SRSVSSGSRSPPRRGKSPAGPARRGRSPPPPPSKGASSPSKKAVQESLVL   100

AT1G16610_P1   HVDSLSRNVNEAHLKEIFGNFGEVIHVEIAMDRAVNLPRGHGYVEFKARA   150
AT1G16610_P2   HVDSLSRNVNEAHLKEIFGNFGEVIHVEIAMDRAVNLPRGHGYVEFKARA   150

AT1G16610_P1   DAEKAQLYMDGAQIDGKVVKATFTLPPRQKVSSPPKPVSAAPKRDAPKSD   200
AT1G16610_P2   DAEKAQLYMDGAQIDGKVVKATFTLPPRQKVSSPPKPVSAAPKRDAPKSD   200

AT1G16610_P1   NAAADAEKDGGPRRPRETSPQRKTGLSPRRRSPLPRRGLSPRRRSPDSPH   250
AT1G16610_P2   NAAADAEKDGGPRRPRER-------LSPRRRSPLPRRGLSPRRRSPDSPH   243

AT1G16610_P1   RRRPGSPIRRRGDTPPRRRPASPSRGRSPSSPPPRRYRSPPRGSPRRIRG   300
AT1G16610_P2   RRRPGSPIRRRGDTPPRRRPASPSRGRSPSSPPPRRYRSPPRGSPRRIRG   293

AT1G16610_P1   SPVRRRSPLPLRRRSPPPRRLRSPPRRSPIRRRSRSPIRRPGRSRSSSIS   350
AT1G16610_P2   SPVRRRSPLPLRRRSPPPRRLRSPPRRSPIRRRSRSPIRRPGRSRSSSIS   343

AT1G16610_P1   PRKGRGPAGRRGRSSSYSSSPSPRRIPRKISRSRSPKRPLRGKRSSSNSS   400
AT1G16610_P2   PRKGRGPAGRRGRSSSYSSSPSPRRIPRKISRSRSPKRPLRGKRSSSNSS   393

AT1G16610_P1   SSSSPPPPPPPRKT                                       414
AT1G16610_P2   SSSSPPPPPPPRKT                                       407
```

VRN5 – AT3G24440

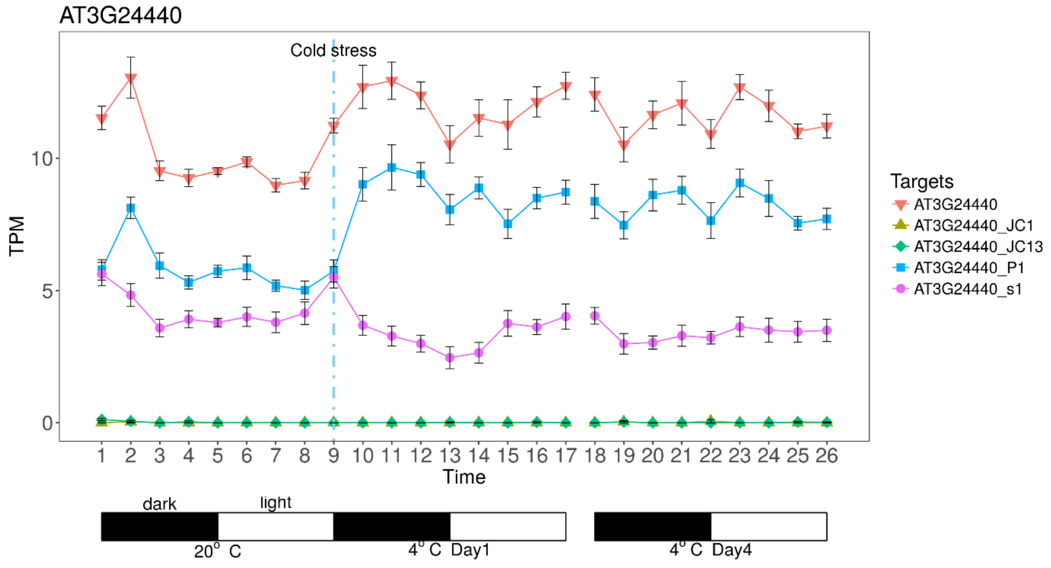


```
AT3G24440_P1  MDSSSTKSKISHSRKTNKKSNKKHESNGKQQQQQDVDGGGGCLRSSWICK  50
AT3G24440_s1  MDSSSTKSKISHSRKTNKKSNKKHESNGKQQQQQDVDGGGGCLRSSWICK  50

AT3G24440_P1  NASCRANVPKEDSFCKRCSCCVCHNFDENKDPSLWLVCEPEKSDDVEFCG  100
AT3G24440_s1  NASCRANVPKEDSFCKRCSCCVCHNFDENKDPSLWLVCEPEKSDDVEFCG  100

AT3G24440_P1  LSCHIECAFREVKVGVIALGNLMKLDGCFCCYSCGKVSQILGCWKKQLVA  150
AT3G24440_s1  LSCHIECAFREVKVGVIALGNLMKLDGCFCCYSCGKVSQILGCWKKQLVA  150

AT3G24440_P1  AKEARRRDGLCYRIDLGYRLLNGTSRFSELHEIVRAAKSMLEDEVGPLDG  200
AT3G24440_s1  AKEARRRDGLCYRIDLGYRLLNGTSRFSELHEIVRAAKSMLEDEVGPLDG  200

AT3G24440_P1  PTARTDRGIVSRLPVAANVQELCTSAIKKAGELSANAGRDLVPAACRFHF  250
AT3G24440_s1  PTARTDRGIVSRLPVAANVQELCTSAIKKAGELSANA--DLVPAACRFHF  248

AT3G24440_P1  EDIAPKQVTLRLIELPSAVEYDVKGYKLWYFKKGEMPEDDLFVDCSRTER  300
AT3G24440_s1  EDIAPKQVTLRLIELPSAVEYDVKGYKLWYFKKGEMPEDDLFVDCSRTER  298

AT3G24440_P1  RMVISDLEPCTEYTFRVVSYTEAGIFGHSNAMCFTKSVEILKPVDGKEKR  350
AT3G24440_s1  RMVISDLEPCTEYTFRVVSYTEAGIFGHSNAMCFTKSVEILKPVDGKEKR  348

AT3G24440_P1  TIDLVGNAQPSDREEKSSISSRFQIGQLGKYVQLAEAQEEGLLEAFYNVD  400
AT3G24440_s1  TIDLVGNAQPSDREEKSSISSRFQIGQLGKYVQLAEAQEEGLLEAFYNVD  398

AT3G24440_P1  TEKICEPPEEELPPRRPHGFDLNVVSVPDLNEEFTPPDSSGGEDNGVPLN  450
AT3G24440_s1  TEKICEPPEEELPPRRPHGFDLNVVSVPDLNEEFTPPDSSGGEDNGVPLN  448

AT3G24440_P1  SLAEADGGDHDDNCDDAVSNGRRKNNNDCLVISDGSGDDTGFDFLMTRKR  500
AT3G24440_s1  SLAEADGGDHDDNCDDAVSNGRRKNNNDCLVISDGSGDDTGFDFLMTRKR  498

AT3G24440_P1  KAISDSNDSENHECDSSSIDDTLEKCVKVIRWLEREGHIKTTFRVRFLTW  550
AT3G24440_s1  KAISDSNDSENHECDSSSIDDTLEKCVKVIRWLEREGHIKTTFRVRFLTW  548

AT3G24440_P1  FSMSSTAQEQSVVSTFVQTLEDDPGSLAGQLVDAFTDVVSTKRPNNGVMT  600
AT3G24440_s1  FSMSSTAQEQSVVSTFVQTLEDDPGSLAGQLVDAFTDVVSTKRPNNGVMT  598

AT3G24440_P1  SH                                                  602
AT3G24440_s1  SH                                                  600
```

SWN - AT4G02020

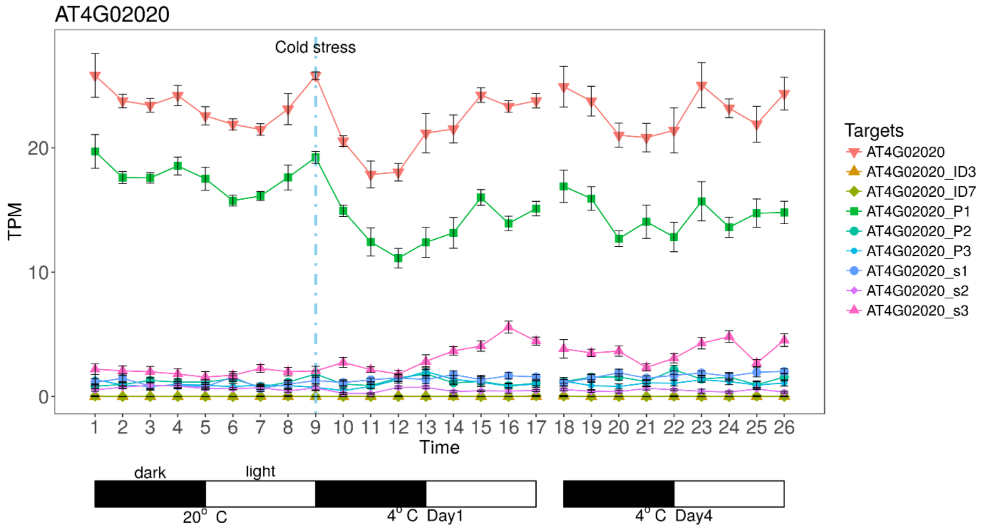


```
AT4G02020_P1  MVTDDSNSSGRIKSHVDDDDDGEEEEDRLEGLENRLSELKRKIQGERVRS  50
AT4G02020_s3  MVTDDSNSSGRI-SHVDDDDDGEEEEDRLEGLENRLSELKRKIQGERVRS  49

AT4G02020_P1  IKEKFEANRKKVDAHVSPFSSAASSRATAEDNGNSNMLSSRMRMPLCKLN  100
AT4G02020_s3  IKEKFEANRKKVDAHVSPFSSAASSRATAEDNGNSNMLSSRMRMPLCKLN  99

AT4G02020_P1  GFSHGVGDRDYVPTKDVISASVKLPIAERIPPYTTWIFLDRNQRMAEDQS  150
AT4G02020_s3  GFSHGVGDRDYVPTKDVISASVKLPIAERIPPYTTWIFLDRNQRMAEDQS  149

AT4G02020_P1  VVGRRQIYYEQHGGETLICSDSEEEPEPEEEKREFSEGEDSIIWLIGQEY  200
AT4G02020_s3  VVGRRQIYYEQHGGETLICSDSEEEPEPEEEKREFSEGEDSIIWLIGQEY  199

AT4G02020_P1  GMGEEVQDALCQLLSVDASDILERYNELKLKDKQNTEEFSNSGFKLGISL  250
AT4G02020_s3  GMGEEVQDALCQLLSVDASDILERYNELKLKDKQNTEEFSNSGFKLGISL  249

AT4G02020_P1  EKGLGAALDSFDNLFCRRCLVFDCRLHGCSQPLISASEKQPYWSDYEGDR  300
AT4G02020_s3  EKGLGAALDSFDNLFCRRCLVFDCRLHGCSQPLISASEKQPYWSDYEGDR  299

AT4G02020_P1  KPCSKHCYLQLKAVREVPETCSNFASKAEEKASEEECSKAVSSDVPHAAA  350
AT4G02020_s3  KPCSKHCYLQLKAVREVPETCSNFASKAEEKASEEECSKAVSSDVPHAAA  349

AT4G02020_P1  SGVSLQVEKTDIGIKNVDSSSGVEQEHGIRGKREVPILKDSNDLPNLSNK  400
AT4G02020_s3  SGVSLQVEKTDIGIKNVDSSSGVEQEHGIRGKREVPILKDSNDLPNLSNK  399

AT4G02020_P1  KQKTAASDTKMSFVNSVPSLDQALDSTKGDQGGTTDNKVNRDSEADAKEV  450
AT4G02020_s3  KQKTAASDTKMSFVNSVPSLDQALDSTKGDQGGTTDNKVNRDSEADAKEV  449

AT4G02020_P1  GEPIPDNSVHDGGSSICQPHHGSGNGAIIIAEMSETSRPSTEWNPIEKDL  500
AT4G02020_s3  GEPIPDNSVHDGGSSICQPHHGSGNGAIIIAEMSETSRPSTEWNPIEKDL  499

AT4G02020_P1  YLKGVEIFGRNSCLIARNLLSGLKTCLDVSNYMRENEVSVFRRSSTPNLL  550
AT4G02020_s3  YLKGVEIFGRNSCLIARNLLSGLKTCLDVSNYMRENEVSVFRRSSTPNLL  549

AT4G02020_P1  LDDGRTDPGNDNDEVPPRTRLFRRKGKTRKLKYSTKSAGHPSVWKRIAGG  600
AT4G02020_s3  LDDGRTDPGNDNDEVPPRTRLFRRKGKTRKLKYSTKSAGHPSVWKRIAGG  599

AT4G02020_P1  KNQSCKQYTPCGCLSMCGKDCPCLTNETCCEKYCGCSKSCKNRFRGCHCA  650
AT4G02020_s3  KNQSCKQYTPCGCLSMCGKDCPCLTNETCCEKYCGCSKSCKNRFRGCHCA  649

AT4G02020_P1  KSQCRSRQCPCFAAGRECDPDVCRNCWVSCGDGSLGEAPRRGEGQCGNMR  700
AT4G02020_s3  KSQCRSRQCPCFAAGRECDPDVCRNCWVSCGDGSLGEAPRRGEGQCGNMR  699

AT4G02020_P1  LLLRQQQRILLGKSDVAGWGAFLKNSVSKNEYLGEYTGELISHHEADKRG  750
AT4G02020_s3  LLLRQQQRILLGKSDVAGWGAFLKNSVSKNEYLGEYTGELISHHEADKRG  749

AT4G02020_P1  KIYDRANSSFLFDLNDQYVLDAQRKGDKLKFANHSAKPNCYAKVMFVAGD  800
AT4G02020_s3  KIYDRANSSFLFDLNDQYVLDAQRKGDKLKFANHSAKPNCYAKVMFVAGD  799

AT4G02020_P1  HRVGIFANERIEASEELFYDYRYGPDQAPVWARKPEGSKKDDSAITHRRA  850
AT4G02020_s3  HRVGIFANERIEASEELFYDYRYGPDQAPVWARKPEGSKKDDSAITHRRA  849

AT4G02020_P1  RKHQSH                                              856
AT4G02020_s3  RKHQSH                                              855
```

VRN2 - AT4G16845

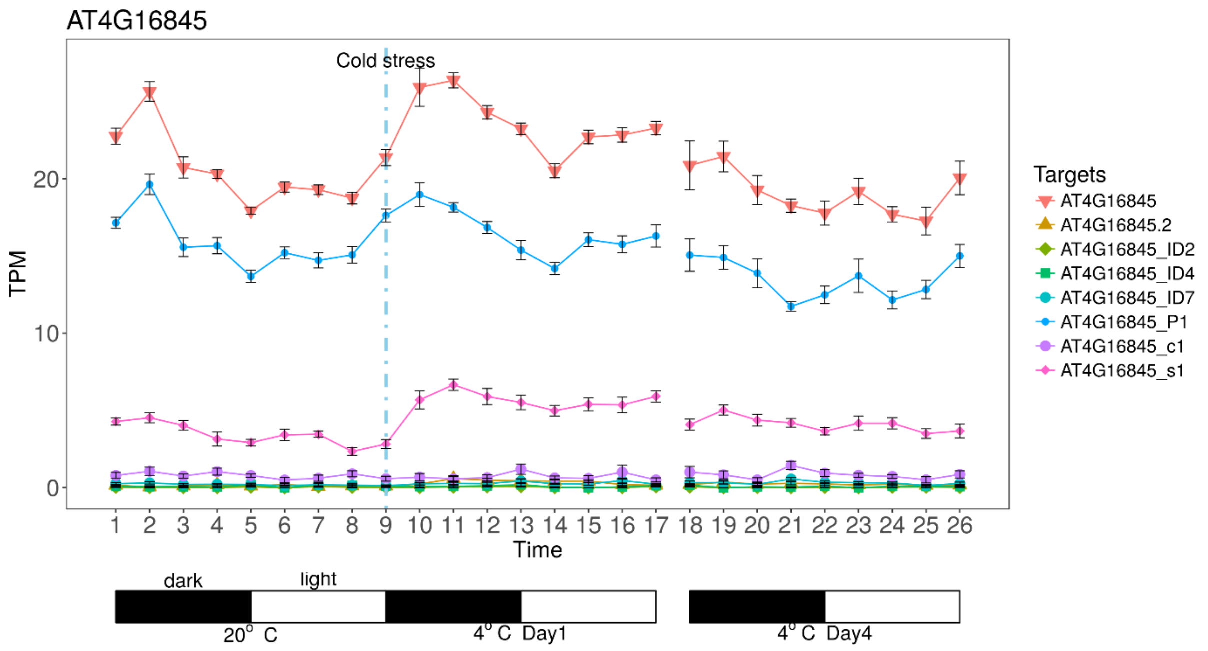


```
AT4G16845_P1  MCRQNCRAKSSPEEVISTDENLLIYCKPVRLYNIFHLRSLGNPSFLPRCL  50
AT4G16845_s1  MCRQNCRAKSSPEEVISTDENLLIYCKPVRLYNIFHLRSLGNPSFLPRCL  50

AT4G16845_P1  NYKIGAKRKRKSRSTGMVVFNYKDCNNTLQRTEVREDCSCPFCSMLCGSF  100
AT4G16845_s1  NYKIGAKRKRKSRSTGMVVFNYKDCNNTLQRTEVREDCSCPFCSMLCGSF  100

AT4G16845_P1  KGLQFHLNSSHDLFEFEFKLLEEYQTVNVSVKLNSFIFEEEGSDDDKFEP  150
AT4G16845_s1  KGLQFHLNSSHDLFEFEFKLLEEYQTVNVSVKLNSFIFEEEGSDDDKFEP  150

AT4G16845_P1  FSLCSKPRKRRQRGGRNNTRRLKVCFLPLDSPSLANGTENGIALLNDGNR  200
AT4G16845_s1  FSLCSKPRKRRQRGGRNNTRRLKVCFLPLDSPSLANGTENGIALLNDGNR  200

AT4G16845_P1  GLGYPEATELAGQFEMTSNIPPAIAHSSLDAGAKVILTTEAVVPATKTRK  250
AT4G16845_s1  GLGYPEATELAGQFEMTSNIPPAIAHSSLDAGAKVILTTEAVVPATKTRK  250

AT4G16845_P1  LSAERSEARSHLLLQKRQFYHSHRVQPMALEQVMSDRDSEDEVDDDVADF  300
AT4G16845_s1  LSAERSEARSHLLLQKRQFYHSHRVQPMALEQVMSDRDSEDEVDDDVADF  300

AT4G16845_P1  EDR--QMLDDFVDVNKDEKQFMHLWNSFVRKQRVIADGHISWACEVFSRF  348
AT4G16845_s1  EDRQLQMLDDFVDVNKDEKQFMHLWNSFVRKQRVIADGHISWACEVFSRF  350

AT4G16845_P1  YEKELHCYSSLFWCWRLFLIKLWNHGLVDSATINNCNTILENCRNTSVTN  398
AT4G16845_s1  YEKELHCYSSLFWCWRLFLIKLWNHGLVDSATINNCNTILENCRNTSVTN  400

AT4G16845_P1  NNNNSVDHPSDSNTNNNNIVDHPNDIKNKNNVDNKDNNSRDK          440
AT4G16845_s1  NNNNSVDHPSDSNTNNNNIVDHPNDIKNKNNVDNKDNNSRDK          442
```

VEL1/VIL2 – AT4G30200

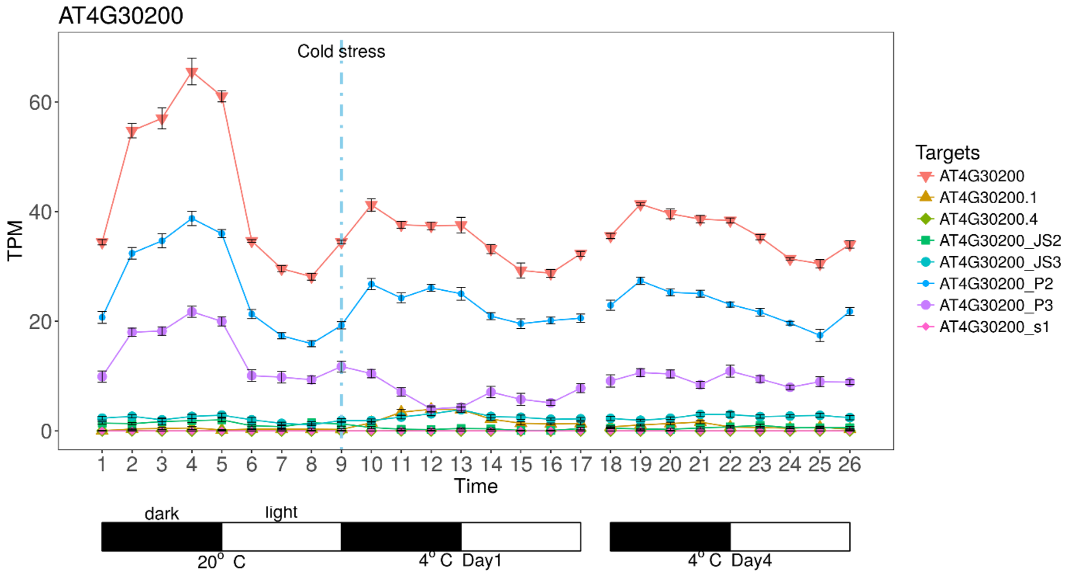


```
AT4G30200_P2  MDSSLDGAAGDSSKCSEMSVDEKRQLVYELSKQSHLAAEVLQAWSRQEIL  50
AT4G30200_P3  MDSSLDGAAGDSSKCSEMSVDEKRQLVYELSKQSHLAAEVLQAWSRQEIL  50

AT4G30200_P2  QILCAEMGKERKYTGLTKVKIIETLLKIVSEKNSGECEGKKRDSDCLPIQ  100
AT4G30200_P3  QILCAEMGKERKYTGLTKVKIIETLLKIVSEKNSGECEGKKRDSDCLPIQ  100

AT4G30200_P2  RNTKRQRKVDNPSRYVIPATNIVTSNNASGSCSSVNTKGESTTIYCKNLA  150
AT4G30200_P3  RNTKRQRKVDNPSRYVIPATNIVTSNNASGSCSSVNTKGESTTIYCKNLA  150

AT4G30200_P2  CRAVLRQEDSFCRRCSCCICRKYDDNKDPSLWLTCSSDPPFEGESCGFSC  200
AT4G30200_P3  CRAVLRQEDSFCRRCSCCICRKYDDNKDPSLWLTCSSDPPFEGESCGFSC  200

AT4G30200_P2  HLECAFNTEKSGLGKDKQSEGCCFYCVSCGKANSLLECWKKQLTIAKETR  250
AT4G30200_P3  HLECAFNTEKSGLGKDKQSEGCCFYCVSCGKANSLLECWKKQLTIAKETR  250

AT4G30200_P2  RVEVLCYRLFLVQKLLKSSTKYRNLCEVVDEAVKTLEADVGPLTGLPMKM  300
AT4G30200_P3  RVEVLCYRLFLVQKLLKSSTKYRNLCEVVDEAVKTLEADVGPLTGLPMKM  300

AT4G30200_P2  GRGIVNRLHSGPDVQKLCSSALESLETIATTPPDVAALPSPRSSKMQQDC  350
AT4G30200_P3  GRGIVNRLHSGPDVQKLCSSALESLETIATTPPDVAALPSPRSSKMQQ--  348

AT4G30200_P2  SYVLSNEISADTATTGSTKIRFEDVNATSLTVVLASNEIPSPPNIVHYSI  400
AT4G30200_P3  ----------DTATTGSTKIRFEDVNATSLTVVLASNEIPSPPNIVHYSI  388

AT4G30200_P2  WHRKVPEKDYPEKSTCTLFIPNTRFVVSGLAPASEYCFKVVSYSGTREMG  450
AT4G30200_P3  WHRKVPEKDYPEKSTCTLFIPNTRFVVSGLAPASEYCFKVVSYSGTREMG  438

AT4G30200_P2  VDEINVLTRSAEEGANCSSAVERSVSPLTNCSTLSSNPSSVEAESNNDYI  500
AT4G30200_P3  VDEINVLTRSAEEGANCSSAVERSVSPLTNCSTLSSNPSSVEAESNNDYI  488

AT4G30200_P2  VPKKPSSKNEDNNSPSVDESAAKRMKRTTDSDIVQIEKDVEQIVLLDDEE  550
AT4G30200_P3  VPKKPSSKNEDNNSPSVDESAAKRMKRTTDSDIVQIEKDVEQIVLLDDEE  538

AT4G30200_P2  QEAVLDKTESETPVVVTTKSLVGNRNSSDASLPITPFRSDEIKNRQARIE  600
AT4G30200_P3  QEAVLDKTESETPVVVTTKSLVGNRNSSDASLPITPFRSDEIKNRQARIE  588

AT4G30200_P2  ISMKDNCNNGDHSANGGTESGLEHCVKIIRQLECSGHIDKNFRQKFLTWY  650
AT4G30200_P3  ISMKDNCNNGDHSANGGTESGLEHCVKIIRQLECSGHIDKNFRQKFLTWY  638

AT4G30200_P2  SLRATSQEIRVVKIFIDTFIDDPMALAEQLIDTFDDRVSIKRSAVGGSGA  700
AT4G30200_P3  SLRATSQEIRVVKIFIDTFIDDPMALAEQLIDTFDDRVSIKRSAVGGSGA  688

AT4G30200_P2  SAVVPSGFCMKLWH                                      714
AT4G30200_P3  SAVVPSGFCMKLWH                                      702
```

HSL1 – AT4G32010

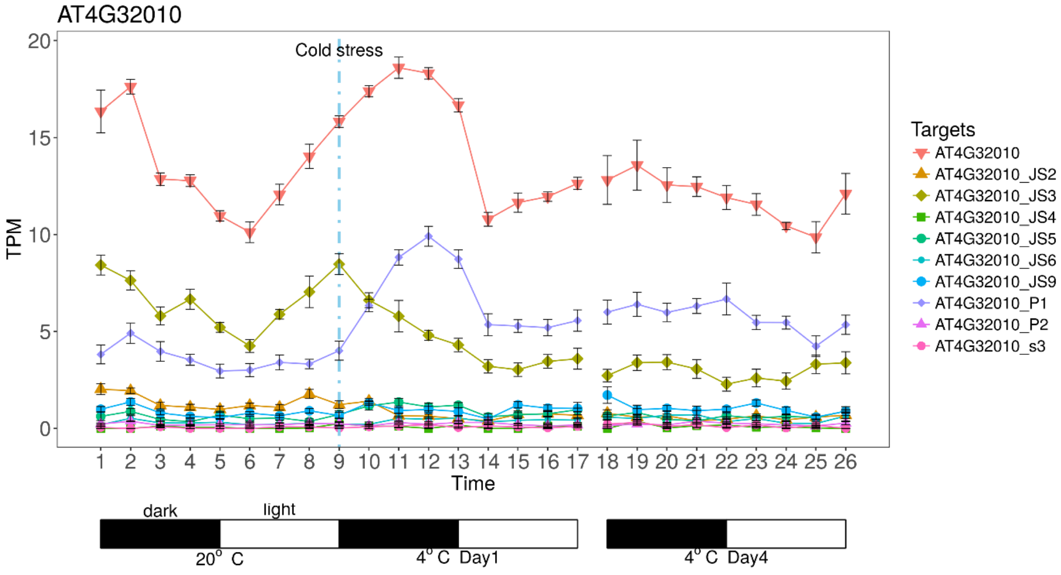


```
AT4G32010_P1   MESIKVCMNALCGAASTSGEWKKGWPMRSGDLASLCDKCGCAYEQSIFCE   50
AT4G32010_JS3  MESIKVCMNALCGAASTSGEWKKGWPMRSGDLASLCDKCGCAYEQSIFCE   50

AT4G32010_P1   VFHAKESGWRECNSCDKRLHCGCIASRFMMELLENGGVTCISCAKKSGLI  100
AT4G32010_JS3  VFHAKESGWRECNSCDKRLHCGCIASRFMMELLENGGVTCISCAKKSGLI  100

AT4G32010_P1   SMNVSHESNGKDFPSFASAEHVGSVLERTNLKHLLHFQRIDPTHSSLQMK  150
AT4G32010_JS3  SMNVSHESNGKDFPSFASAEHVGSVLERTNLKHLLHFQRIDPTHSSLQMK  150

AT4G32010_P1   QEESLLPSSLDALRHKTERKELSAQPNLSISLGPTLMTSPFHDAAVDDRS  200
AT4G32010_JS3  QEESLLPSSLDALRHKTERKELSAQPNLSISLGPTLMTSPFHDAAVDDRS  200

AT4G32010_P1   KTNSIFQLAPRSRQLLPKPANSAPIAAGMEPSGSLVSQIHVARPPPEGRG  250
AT4G32010_JS3  KTNSIFQLAPRSRQLLPKPANSAPIAAGMEPSGSLVSQIHVARPPPEGRG  250

AT4G32010_P1   KTQLLPRYWPRITDQELLQLSGQYPHLSNSKIIPLFEKVLSASDAGRIGR  300
AT4G32010_JS3  KTQLLPRYWPRITDQELLQLSGH----SNSKIIPLFEKVLSASDAGRIGR  296

AT4G32010_P1   LVLPKACAEAYFPPISLPEGLPLKIQDIKGKEWVFQFRFWPNNNSRMYVL  350
AT4G32010_JS3  LVLPKACAEAYFPPISLPEGLPLKIQDIKGKEWVFQFRFWPNNNSRMYVL  346

AT4G32010_P1   EGVTPCIQSMQLQAGDTVTFSRTEPEGKLVMGYRKATNSTATQMFKGSSE  400
AT4G32010_JS3  EGVTPCIQSMQLQAGDTVTFSRTEPEGKLVMGYRKATNSTATQMFKGSSE  396

AT4G32010_P1   PNLNMFSNSLNPGCGDINWSKLEKSEDMAKDNLFLQSSLTSARKRVRNIG  450
AT4G32010_JS3  PNLNMFSNSLNPGCGDINWSKLEKSEDMAKDNLFLQSSLTSARKRVRNIG  446

AT4G32010_P1   TKSKRLLIDSVDVLELKITWEEAQELLRPPQSTKPSIFTLENQDFEEYDE  500
AT4G32010_JS3  TKSKRLLIDSVDVLELKITWEEAQELLRPPQSTKPSIFTLENQDFEEYDE  496

AT4G32010_P1   PPVFGKRTLFVSRQTGEQEQWVQCDACGKWRQLPVDILLPPKWSCSDNLL  550
AT4G32010_JS3  PPVFGKRTLFVSRQTGEQEQWVQCDACGKWRQLPVDILLPPKWSCSDNLL  546

AT4G32010_P1   DPGRSSCSAPDELSPREQDTLVRQSKEFKRRRLASSNEKLNQSQDASALN  600
AT4G32010_JS3  DPGRSSCSAPDELSPREQDTLVRQSKEFKRRRLASSNEKLNQSQDASALN  596

AT4G32010_P1   SLGNAGITTTGEQGEITVAATTKHPRHRAGCSCIVCSQPPSGKGKHKPSC  650
AT4G32010_JS3  SLGNAGITTTGEQGEITVAATTKHPRHRAGCSCIVCSQPPSGKGKHKPSC  646

AT4G32010_P1   TCTVCEAVKRRFRTLMLRKRNKGEAGQASQQAQSQSECRDETEVESIPAV  700
AT4G32010_JS3  TCTVCEAVKRRFRTLMLRKRNKGEAGQASQQAQSQSECRDETEVESIPAV  696

AT4G32010_P1   ELAAGENIDLNSDPGASRVSMMRLLQAAAFPLEAYLKQKAISNTAGEQQS  750
AT4G32010_JS3  ELAAGENIDLNSDPGASRVSMMRLLQAAAFPLEAYLKQKAISNTAGEQQS  746

AT4G32010_P1   SDMVSTEHGSSSAAQETEKDTTNGAHDPVN                      780
AT4G32010_JS3  SDMVSTEHGSSSAAQETEKDTTNGAHDPVN                      776
```

EMF2 – AT5G51230

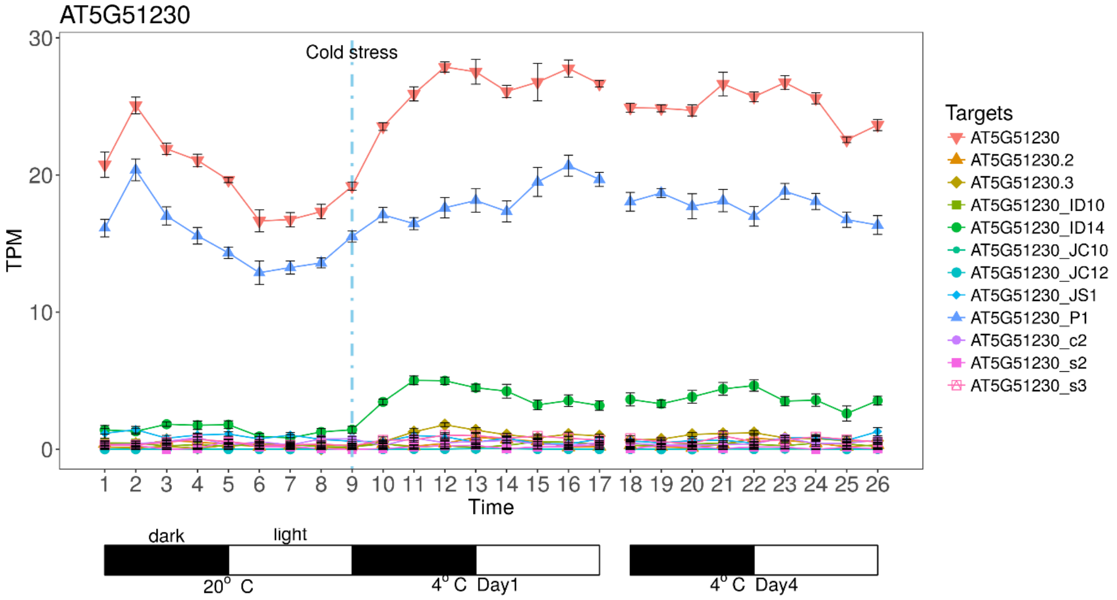


```
AT5G51230_P1    MPGIPLVSRETSSCSRSTEQMCHEDSRLRISEEEEIAAEESLAAYCKPVE   50
AT5G51230_ID14  MPGIPLVSRETSSCSRSTEQMCHEDSRLRISEEEEIAAEESLAAYCKPVE   50

AT5G51230_P1    LYNIIQRRAIRNPLFLQRCLHYKIEAKHKRRIQMTVFLSGAIDAGVQTQK   100
AT5G51230_ID14  LYNIIQRRAIRNPLFLQRCLHYKIEAKHKRRIQMTVFLSGAIDAGVQTQK   100

AT5G51230_P1    LFPLYILLARLVSPKPVAEYSAVYRFSRACILTGGLGVDGVSQAQANFLL   150
AT5G51230_ID14  LFPLYILLARLVSPKPVAEYSAVYRFSRACILTGGLGVDGVSQAQANFLL   150

AT5G51230_P1    PDMNRLALEAKSGSLAILFISFAGAQNSQFGIDSGKIHSGNIGGHCLWSK   200
AT5G51230_ID14  PDMNRLALEAKSGSLAILFISFAGAQNSQFGIDSGKIHSGNIGGHCLWSK   200

AT5G51230_P1    IPLQSLYASWQKSPNMDLGQRVDTVSLVEMQPCFIKLKSMSEEKCVSIQV   250
AT5G51230_ID14  IPLQSLYASWQKSPNMDLGQRVDTVSLVEMQPCFIKLKSMSEEKCVSIQV   250

AT5G51230_P1    PSNPLTSSSPQQVQVTISAEEVGSTEKSPYSSFSYNDISSSSLLQIIRLR   300
AT5G51230_ID14  PSNPLTSSSPQQVQVTISAEEVGSTEKSPYSSFSYNDISSSSLLQIIRLR   300

AT5G51230_P1    TGNVVFNYRYYNNKLQKTEVTEDFSCPFCLVKCASFKGLRYHLPSTHDLL   350
AT5G51230_ID14  TGNVVFNYRYYNNKLQKTEVTEDFSCPFCLVKCASFKGLRYHLPSTHDLL   350

AT5G51230_P1    NFEFWVTEEFQ--------------AVNVSLKTETMISKVNEDDVDPKQQ   386
AT5G51230_ID14  NFEFWVC-SFKIQLTCLIFFFYFVGATNLLYLVR----------------   383

AT5G51230_P1    TFFFSSKKFRRRRQKSQVRSSRQGPHLGLGCEVLDKTDDAHSVRSEKSRI   436
AT5G51230_ID14  --------------------------------------------------   383

AT5G51230_P1    PPGKHYERIGGAESGQRVPPGTSPADVQSCGDPDYVQSIAGSTMLQFAKT   486
AT5G51230_ID14  --------------------------------------------------   383

AT5G51230_P1    RKISIERSDLRNRSLLQKRQFFHSHRAQPMALEQVLSDRDSEDEVDDDVA   536
AT5G51230_ID14  --------------------------------------------------   383

AT5G51230_P1    DFEDRRMLDDFVDVTKDEKQMMHMWNSFVRKQRVLADGHIPWACEAFSRL   586
AT5G51230_ID14  --------------------------------------------------   383

AT5G51230_P1    HGPIMVRTPHLIWCWRVFMVKLWNHGLLDARTMNNCNTFLEQLQI        631
AT5G51230_ID14  ---------------------------------------------        383
```

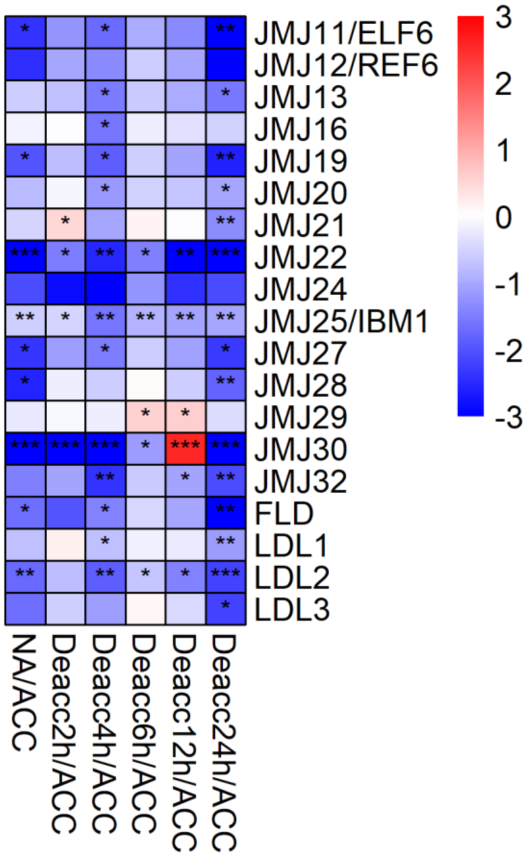

Supplemental Figure 3: Expression changes of genes encoding JUMONJI-type and LSD1-type histone demethylases at non-acclimated conditions (NA) and after 2 h, 4 h, 6 h, 12 h and 24 h of deacclimation (Deacc). Gene expression is presented as log2 fold change to cold acclimated conditions (ACC) (Suppl. Table 7). The scale of log2 fold changes ranges from -3 (blue) to 3 (red) with a median of 0 (white). Significance levels are indicated relative to ACC: ***, $P < 0.001$; **, $P < 0.01$; *, $P < 0.05$ *.

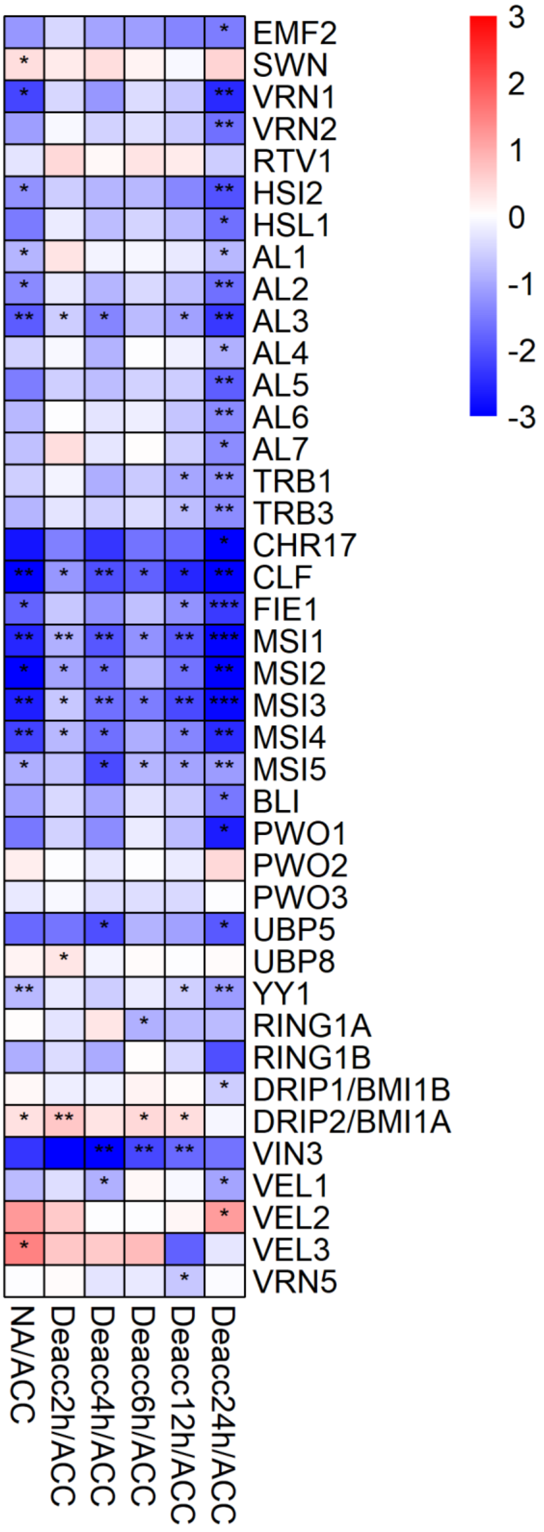

Supplemental Figure 4: Expression changes of genes encoding proteins of the polycomb group (Pc-G) family at non-acclimated conditions (NA) and after 2 h, 4 h, 6 h, 12 h and 24 h of deacclimation (Deacc). Gene expression is presented as log2 fold change to cold acclimated conditions (ACC) (Suppl. Table 7). The scale of log2 fold changes ranges from -3 (blue) to 3 (red) with a median of 0 (white). Significance levels are indicated relative to ACC: ***, $P < 0.001$; **, $P < 0.01$; *, $P < 0.05$ *.

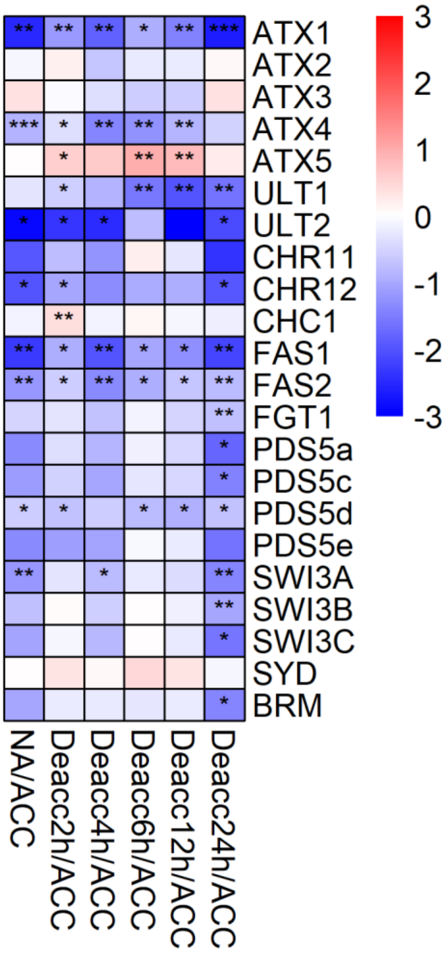

Supplemental Figure 5: Expression changes of genes encoding proteins of the trithorax group (Trx-G) at non-acclimated conditions (NA) and after 2 h, 4 h, 6 h, 12 h and 24 h of deacclimation (Deacc). Gene expression is presented as log2 fold change to cold acclimated conditions (ACC) (Suppl. Table 7). The scale of log2 fold changes ranges from -3 (blue) to 3 (red) with a median of 0 (white). Significance levels are indicated relative to ACC: ***, *P* < 0.001; **, *P* < 0.01; *, *P* < 0.05 *.

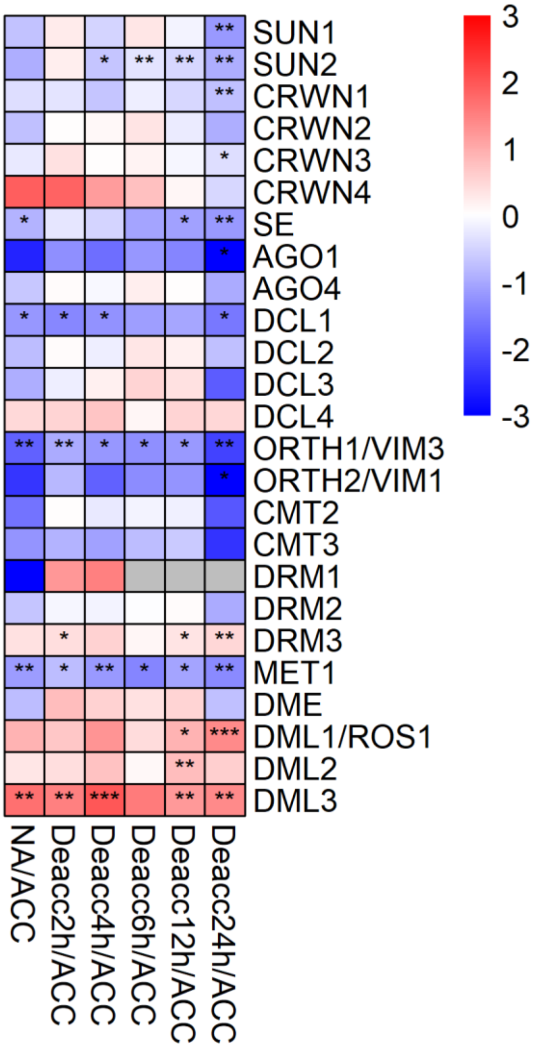

Supplemental Figure 6: Expression changes of genes encoding proteins acting in chromosome-nuclear envelope (Chr-NE) interactions, RNA interference and methylation at non-acclimated conditions (NA) and after 2 h, 4 h, 6 h, 12 h and 24 h of deacclimation (Deacc). Gene expression is presented as log2 fold change to cold acclimated conditions (ACC) (Suppl. Table 7). The scale of log2 fold changes ranges from -3 (blue) to 3 (red) with a median of 0 (white). Significance levels are indicated relative to ACC: ***, $P < 0.001$; **, $P < 0.01$; *, $P < 0.05$ *.

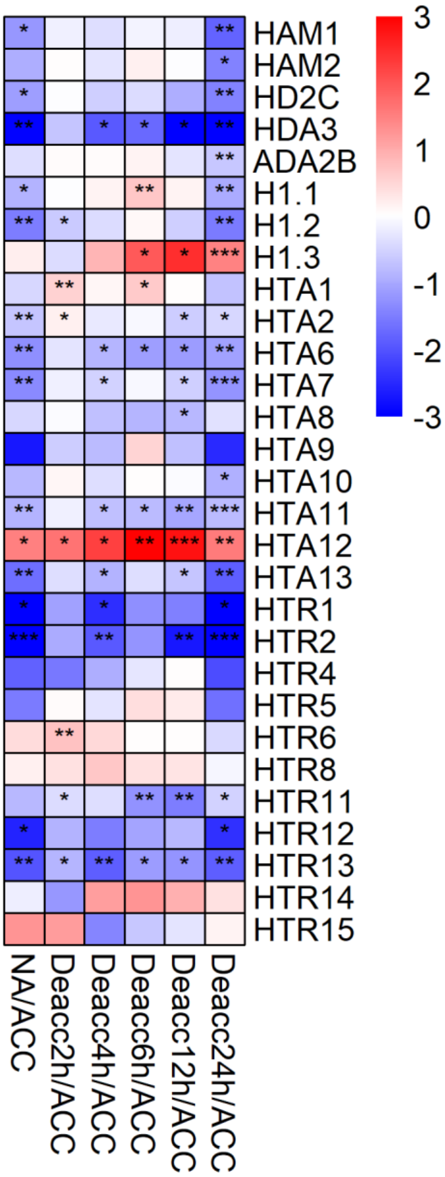

Supplemental Figure 7: Expression changes of genes encoding histone acetyltransferases (HAC), deacetylases (HDAC) or histone variants at non-acclimated conditions (NA) and after 2 h, 4 h, 6 h, 12 h and 24 h of deacclimation (Deacc). Gene expression is presented as log2 fold change to cold acclimated conditions (ACC) (Suppl. Table 7). The scale of log2 fold changes ranges from -3 (blue) to 3 (red) with a median of 0 (white). Significance levels are indicated relative to ACC: ***, *P* < 0.001; **, *P* < 0.01; *, *P* < 0.05 *.
